# Navigating prokaryotic viral genome analysis from metagenomic data

**DOI:** 10.1128/msystems.01249-25

**Published:** 2026-04-21

**Authors:** Almut Werner, Cynthia M. Chibani, Ruth A. Schmitz

**Affiliations:** 1 Institute for General Microbiology, Christian-Albrechts-University 9179, Kiel, Germany; Medizinische Universitat Graz, Graz, Austria

**Keywords:** viromics, bioinformatics methods, computational biology, DNA viruses, metagenomics, archaea, bacteria

## Abstract

Viruses play crucial roles in microbial ecosystems, yet viromic analysis remains challenging due to the field’s complexity and rapid evolution. This minireview supports non-specialists through the evolving landscape of viromics, focusing on the analysis of bacterial and archaeal DNA viruses from metagenomic data. We address major challenges, including viral diversity, methodological biases, and the overwhelming array of available tools and pipelines. While describing a typical viromic workflow, we provide users with background information for each of the steps from data acquisition, preprocessing, and quality control to viral characterization and common downstream analyses. The included references and resources will provide users with the information needed to confidently start their own virome analysis.

## INTRODUCTION

Viruses are major components of microbial ecosystems, where they influence host populations through lysis, reshape microbial metabolism via auxiliary metabolic genes (AMGs) carried by proviruses, and facilitate horizontal gene transfer (1–5). These processes contribute to community structure, evolutionary dynamics, and global biogeochemical cycles. Over the past decade, advances in sequencing and computational methods have led to a surge in virus studies, uncovering vast viral diversity and revealing viruses as major players in microbial ecology across nearly every biome (6–10).

Viromics, the study of viral genomes recovered directly from metagenomic data sets, has enabled researchers to investigate viral diversity, distribution, ecological roles, evolutionary patterns, and interactions with microbial hosts at genome resolution. Despite this progress, analyzing viral sequences from metagenomic data remains technically challenging. Unlike bacteria and archaea, viruses do not possess universal marker genes. They often show high sequence divergence, have small genome sizes, and frequently assemble into short or fragmented contigs, especially when working with complex environmental samples (11). These difficulties are further amplified by biases introduced during sample processing steps such as viral enrichment, filtration, and amplification. Such methods can selectively favor certain viral groups while underrepresenting others, ultimately skewing the apparent community composition (12).

In addition to these biological and technical challenges, the growing number of available tools, the continuous developments in concepts and analytical methods, and the significant computational demands can make viromics difficult to approach. This complexity poses a barrier to researchers who lack formal training. Selecting suitable tools and interpreting their results can be difficult, particularly given the diversity of available methods and evaluation criteria.

This guide is designed for users without prior experience in viromics, as well as for those looking to revisit or strengthen their understanding of specific parts of the analysis. Rather than functioning as a tutorial for individual software tools, we emphasize broader decision points throughout a typical workflow. By combining practical advice with conceptual context, this guide is designed to help users initiate viromics projects with greater clarity and confidence while also deepening their understanding of the possibilities and tradeoffs that shape viral metagenomic research.

We also aim to serve as a reference for researchers and reviewers seeking to understand the assumptions and limitations behind viromics analyses in general. We focus on DNA viruses infecting bacterial and archaeal hosts (bacteriophages and archaeal viruses are referred to as viruses in our guide) and provide a clear overview of each major step in the viromics workflow. To help users make informed decisions, we also discuss the purpose, capabilities, and limitations of commonly used tools, emphasizing potential biases introduced during analysis where applicable. Throughout the guide, we provide links to original tool publications, benchmarking studies, and foundational literature in the field, as well as options for follow-up analyses and additional reading for deeper exploration of viral ecology, function, and evolution. While comparing three widely used viromics pipelines in detail (MVP [13], ViroProfiler [14], and ViWrap [15]), we offer practical guidance on how to choose between them based on study goals and available resources. In addition, we compiled a structured and comprehensive overview of tools available for each step of the workflow to assist users in exploring options that fit their specific needs and data sets (Tables S1 and S2). Finally, we compiled common issues and problematic results that can arise during any of the detailed workflow steps while also offering potential explanations and suggested next steps (Table S3). Together, this guide provides users with all the essential components to start their own viromic analysis.

## VIROMICS WORKFLOW

**Fig 1:**
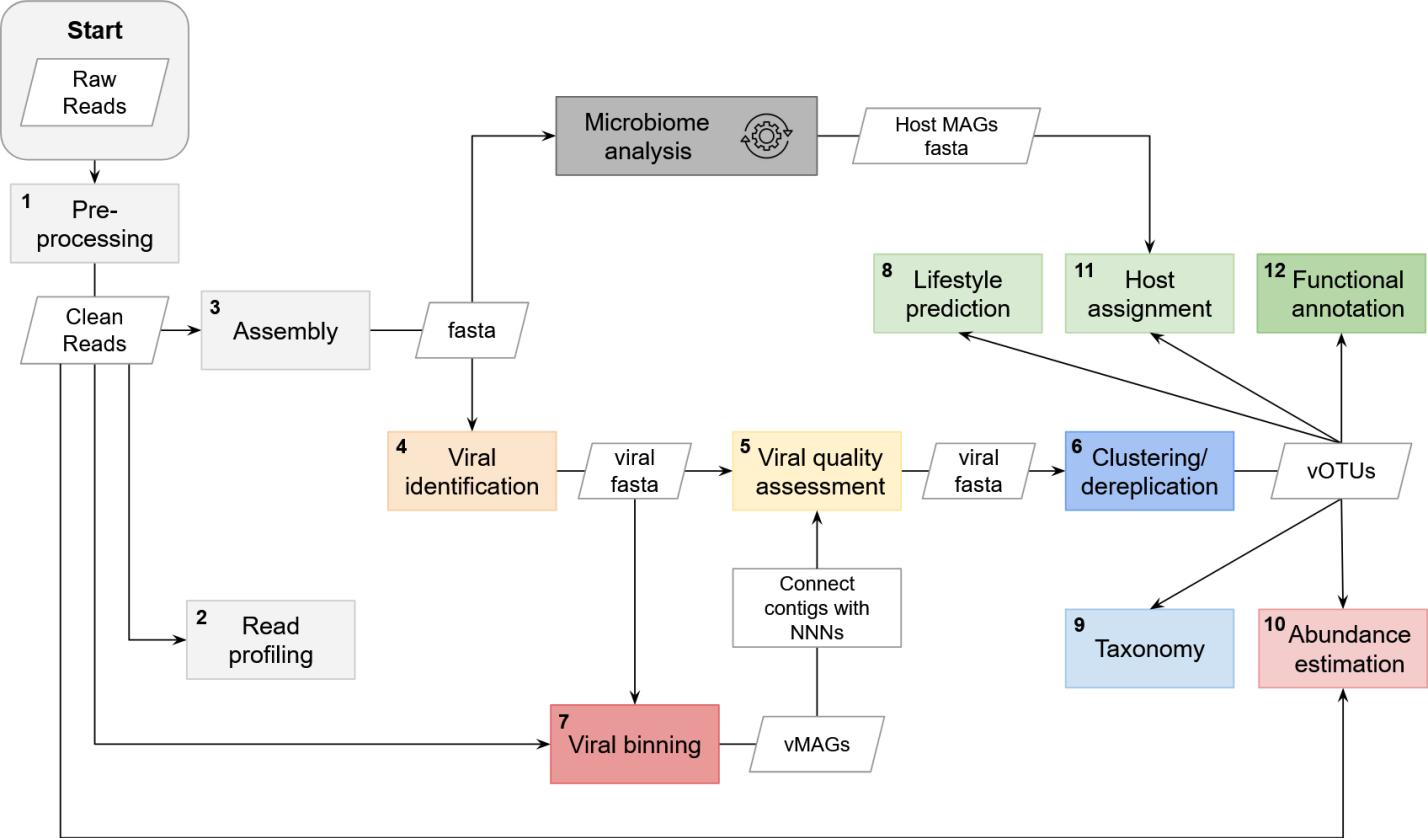
Workflow describing a viromics analysis. Different steps are indicated by boxes with the numbers corresponding to the section order of this paper, while input and output are represented by rhomboid shapes. Pre-processing (step 1 [Supplemental material]) is essential to perform on raw reads. Steps 2 and 5 (Supplemental material) are optional but recommended. Clustering/dereplication (step 6) is essential to reduce the amount of data and cluster viral genomes into vOTUs. Viral binning (step 7) is optional, since it is not yet widely spread and implemented in most pipelines. Steps 8–12 are optional downstream analyses and can be performed on viral sequences, vMAGs, or vOTUs depending on user preferences and research questions. The analysis of prokaryotic hosts from the same metagenomic sample depends on data availability and experimental design. Deviations from this workflow due to problematic results are described in Table S3.

A typical viromic workflow involves several key steps to ensure comprehensive characterization of viral samples (Fig. 1). The process begins with sample collection, preparation, and sequencing (step 0 [Supplemental material]), after which the resulting data undergoes preprocessing to remove low-quality reads and contaminants (step 1 [Supplemental material]). Optionally, a quick taxonomic overview can be generated from clean reads during read profiling (step 2 [Supplemental material]). During assembly, clean reads are combined into contigs (step 3 [Supplemental material]), which are then used for viral identification (step 4). A virus-specific quality control step (step 5 [Supplemental material]) is recommended to ensure accurate data for further analysis of the contigs, or to re-evaluate processed data later on. The final set of viral contigs can be used for clustering (step 6), which results in viral operational taxonomic units (vOTUs), or optional binning (step 7), resulting in viral metagenome-assembled genomes (vMAGs). It is also possible to cluster concatenated vMAGs if required (e.g., in case of high data fragmentation). The following steps can be performed on the resulting viral collection based on user preferences (viral sequences, vMAGs, or vOTUs): lifestyle prediction (step 8), taxonomy (step 9 [Supplemental material]), abundance estimation (step 10 [Supplemental material]), host assignment (step 11), functional annotation (step 12 [Supplemental material]), and other downstream analyses. It is important to note that this workflow is non-linear. To help guide the user through the decision process, we have summarized all discussed tools and their capabilities (Tables S1 and S2). Table S1 not only outlines how each tool operates but also highlights key workflow requirements like metagenome compatibility or notable restrictions. We also provide an overview of common issues and possible troubleshooting approaches (Table S3).

### Step 4: viral identification from assembled contigs

**Fig 2:**
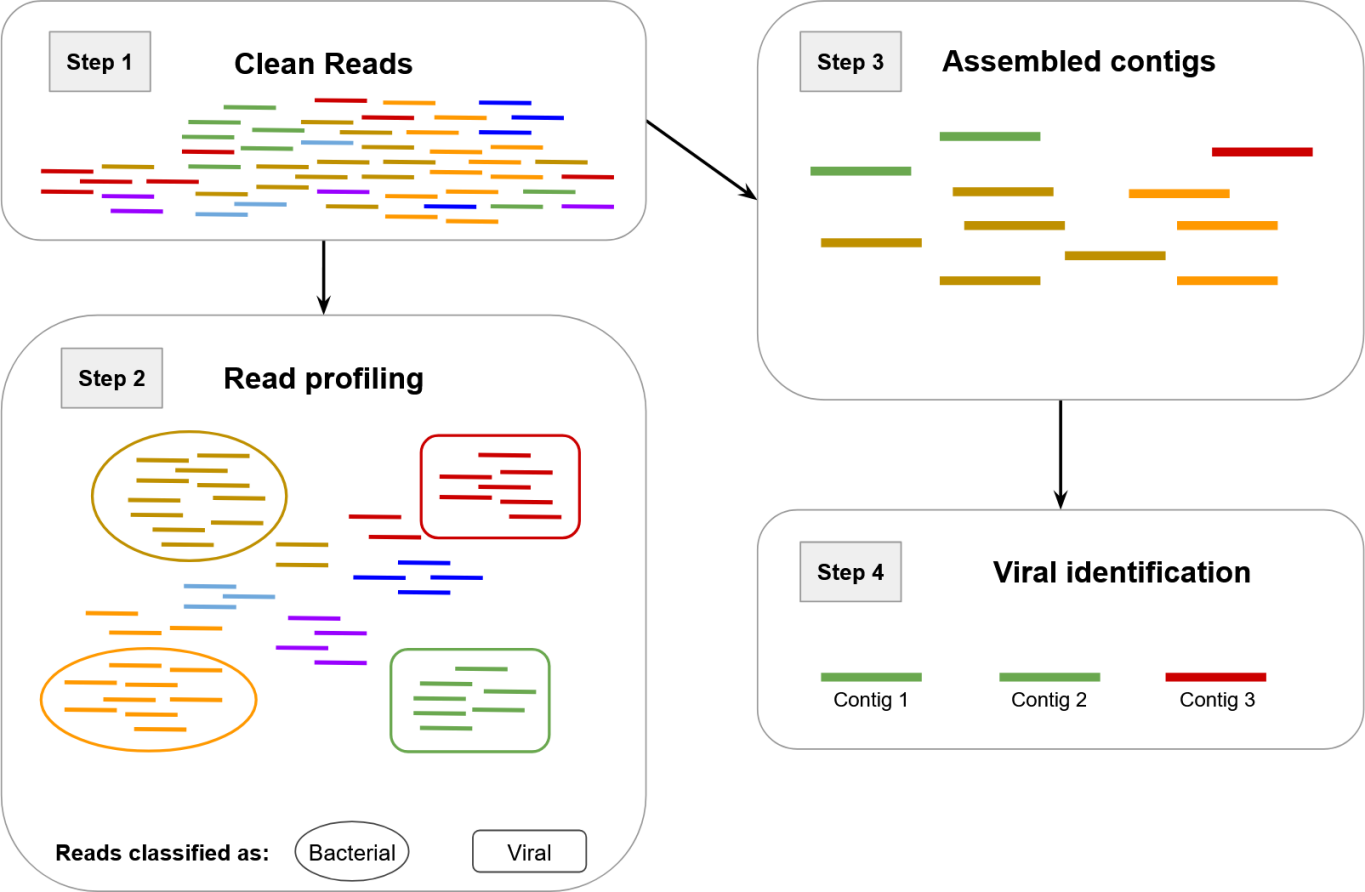
Schematic representation showing an example analysis. Clean reads of different genomes indicated by color (obtained in step 1 [Supplemental material]) are partially classified into viral and bacterial families (step 2 [Supplemental material]). Afterward, reads are assembled into contigs (viral and non-viral), with not all reads resulting in assemblies (step 3 [Supplemental material]). Contigs are then subjected to viral identification (step 4).

While metagenomic data sets enable unique analysis approaches since both host and virus are sequenced together, viruses can only be predicted *in silico*. In addition to inferring viral signals from reads (step 2 [Supplemental material]), viruses can also be predicted from contigs (Fig. 2). While accurate results are essential for meaningful downstream analysis, they can vary significantly between tools due to different approaches, training data, and use case optimizations. This affects a tool’s ability to process different types and lengths of viruses and integrated proviruses. It can additionally lead to biases toward certain habitats, sample types (e.g., isolates), or taxa (virus or host), including not being able to identify certain viral taxa or excluding them entirely (16, 17). Therefore, being mindful of the data set used for benchmarking is important when comparing the performance of different tools, since using sequences belonging to the training data of a tool leads to an inflated positive performance (18). Even though combining tools to maximize the amount of recovered viruses by reducing false negatives (sequences wrongly identified as non-viral) is a popular strategy, it will also lead to an aggregation of false positives (sequences wrongly identified as viral) and should be done cautiously (19). Additionally, there is the common misconception that if two tools report a low likelihood for a sequence to be viral, the probabilities “add up.” In reality, this only means that the two tools agree on the sequence having a low likelihood to be viral (19). One of the causes for false positives lies in suboptimal training or reference data derived from public databases. Many sequences are not scanned and filtered for viral contamination before database submission, which leads to co-sequenced or integrated viruses being submitted and falsely attributed to their hosts (19).

Viral identification tools can be categorized into two types: alignment-free and homology-based models. Homology-based models compare part of the sequence to reference databases and are therefore more prone to misclassifications due to faulty references. Additionally, databases are strongly biased toward viruses with clinical relevance or from well-studied hosts, viruses that have been cultivated, as well as dsDNA viruses, since they are targeted by sequencing efforts (18). Apart from BLASTing (20) the whole sequence to a reference database (e.g., MetaPhinder [21]), common approaches also include using hidden Markov models (HMMs) for virus-associated genes (e.g., Phigaro [22], VIBRANT [23], viralVerify [24], VirSorter [25], and VirSorter2 [26]). A subset of these tools achieves this with the help of different machine learning approaches. Alignment-free models are more independent of databases, although databases and references are still needed to establish training data or underlying knowledge about virus-specific genome signatures. In theory, they are therefore more fit to identify previously unknown viruses dissimilar to known ones, as well as short contigs (≤5 kb) containing few distinctly viral genes that are challenging to classify correctly (19). All current alignment-free models use different machine learning approaches based on, e.g., k-mer motifs (like DeepVirFinder [27], PPR-Meta [28], or VirFinder [29]), or long short-term memory networks (e.g., Seeker [30]); and there are tools combining alignment-free with marker-based approaches (geNomad [31]). However, alignment-free models in particular tend to overestimate the amount of viral sequences. We thus do not recommend using alignment-free models at their current stage, especially if the results are not verified using other methods (including manual screening).

While the increase in reference data and more sensitive tools accelerates the identification of novel viruses, predictions should be experimentally validated when possible, as the viability of a computationally identified virus cannot be guaranteed using predictions only (regardless of the identification method). This holds especially true in the case of integrated proviruses, which can be domesticated (and thus functionally inactivated) by hosts (32). To our knowledge, no tool dedicated to evaluating the viability of a predicted virus (be it integrated or free) exists yet. However, it is possible to make approximations by comparing gene content and organization to reference viruses.

For benchmarks of common tools, see references 16, 19, 31.

### Step 6: viral clustering and dereplication

**Fig 3:**
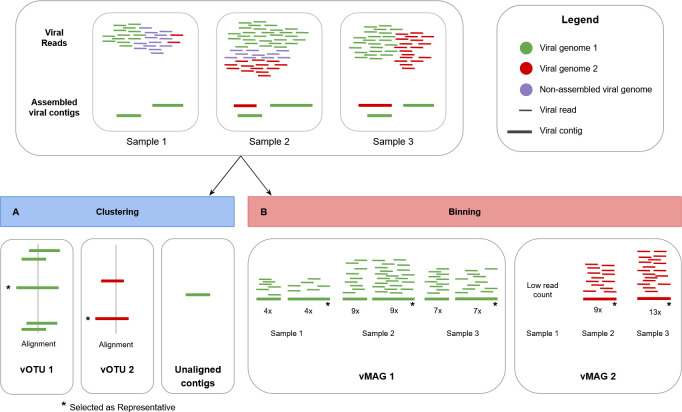
Schematic overview and visual representation of the steps leading to vOTUs (step 10 [Supplemental material]) and vMAGs (step 7). In general, for vOTUs, you capture viral contigs that align. In vMAGs, you capture viral contigs that are co-abundant, originating from the same virus but might not align. Viral binning tools leverage co-abundance patterns and additional metrics not visualized here. In this example, we illustrate (**A**) two vOTU species resulting from clustering all predicted viral contigs from three different samples based on sequence similarity and (**B**) two vMAGs resulting from binning vOTU representatives and the unaligned contig across the three samples based on abundance information (read mapping). vMAG1 is the result of the vOTU representative and one unaligned contig originating from the same virus (green), while vMAG2 is one single contig representing one virus (red).

Naturally, samples contain multiple viral genotypes, and due to aforementioned reasons (step 3 [Supplemental material[), assemblies often produce fragmented genomes. However, downstream analyses often aim to characterize the community at the species level or higher due to computational limitations. To reduce the amount of data, viral genomes (contigs or concatenated viral bins [step 7]) are dereplicated by clustering them into viral operational taxonomic units (vOTUs) that typically serve as a proxy for species-level groups, but other taxonomic levels are possible as well (Fig. 3). Clustering thresholds are based on average nucleotide identity (ANI) and genome coverage. Commonly adopted standards follow minimum information about an uncultivated virus genome (MIUViG [17]), where vOTUs are clustered at **≥**95% ANI and **≥**85% genome coverage relative to the shorter sequence. Additionally, International Committee on Taxonomy of Viruses (ICTV) guidelines (33) recommend taxon demarcation criteria depending on the viral family (e.g., <31% amino acid identity in the L protein of different genera of the family *Aliusviridae* [34, 35]). A long and high-quality sequence from the cluster is then automatically or manually selected as a vOTU representative. Since downstream analyses often focus on these cluster representatives, it is advisable to choose carefully.

Tools that cluster input based on ANI and genome coverage differ in their methods and, thus, in performance and precision. dRep (36) combines a slow but accurate ANI calculation (gANI [37]) with a fast but less precise genome distance estimation (Mash [38]) to optimize performance. It was originally developed for species-level dereplication of archaeal/bacterial genomes and metagenome-assembled genomes (MAGs) but was recently applied using parameters tailored to viral data sets in workflows like ViWrap.

MMseqs2 (39), a general-purpose sequence clustering suite, uses fast k-mer-based prefiltering and sparse indexing for its clustering workflows, which bypasses computationally expensive alignments for dissimilar sequences. It is widely adopted in viral metagenomics for dereplicating large-scale viromes. The parameters are freely adjustable, while workflows like easy-linclust enable rapid processing of millions of sequences.

Vclust is a dedicated viral clustering tool that combines fast k-mer-based prefiltering with Lempel-Ziv-based ANI estimation through LZ-ANI and hierarchical clustering (40). It is fully customizable for different ANI metrics (ANI, global ANI, and total ANI, similar to VIRIDIC’s intergenomic similarity [41]), offering species-level resolution. Vclust is specifically optimized for large-scale data sets while maintaining high clustering accuracy and sensitivity. Its design allows accurate clustering of complete, partial, and circularly permuted viral genomes and remains robust even when dealing with highly fragmented metagenomic data. vConTACT2 (42) follows a different approach by clustering viral genomes based on shared gene content. It constructs a gene-sharing network where viral genomes are connected if they share a significant number of clusters. While only offering genus-level taxonomic resolution, vConTACT2 is particularly useful for novel or unclassified viruses where sequence-based methods may fall short.

Across all tools, the choice of clustering method depends on data set size, genome completeness, and desired taxonomic resolution. Manual curation is often necessary when selecting vOTU representatives, especially if other cluster members are of higher quality or derived from isolates. Vclust and MMseqs2 are currently favored for their combination of speed, accuracy, and ability to handle complex viral data sets. The combination of multiple tools optimized for different taxonomic levels (e.g., species and genus) is common as well.

For a benchmark of different clustering tools, see reference 40.

### Step 7: viral binning

Viral binning is an emerging strategy in viromics that groups contigs originating from the same viral genome into a viral metagenome assembled genome (vMAG) using read coverage patterns and other metrics (Fig. 3). Although binning is standard in bacterial and archaeal genome studies, it is still not widely implemented in viromics. The common assumption that a single contig represents a complete viral genome is flawed and can lead to overestimation of viral diversity, particularly in samples with low abundance viruses or incomplete assemblies (18).

Short-read sequencing generates highly fragmented assemblies (43, 44). Since most viral genomes fall within the 7–20 kb range, and some can extend beyond 2,000 kb as seen in giant viruses (45), multiple contigs may belong to the same viral genome. Without proper binning, these fragmented sequences are either misclassified as separate viruses, as low-quality viruses, or excluded entirely.

Long read sequencing technologies offer a partial solution, since long reads can recover entire viral genomes in a single sequencing pass (46), which is especially effective for small to mid-sized viruses. However, they often contain errors (step 3, Supplementary) and may truncate during sequencing, particularly for very large viral genomes. Therefore, fragmentation remains common also with long-read sequencing approaches, due to biological complexity, low sequencing depth, or larger viral genomes.

As a result, bins containing only one or two contigs should not be discarded. While such bins would typically be excluded in bacterial metagenomics, in viromics, they may still represent complete viral genomes. Even a single contig can still be informative and may fully capture a viral population.

Several computational tools have recently emerged to address the unique challenges of viral binning in metagenomics. vRhyme (47, 48) uses supervised machine learning and coverage effect size comparisons to construct vMAGs, addressing high viral diversity and fragmentation. CoCoNet (49) leverages deep learning to model k-mer composition and abundance profiles to estimate whether contigs originate from the same viral genome. VAMB (50) integrates deep learning-based binning with taxonomic clustering and host-virus association analysis, enabling large-scale recovery of viral genomes. COBRA (51) extends and joins contigs directly producing more complete and contiguous viral genomes than binning alone. In direct comparisons, COBRA outperformed vRhyme, CoCoNet, and MetaBAT2 (52) (specifically used for MAG binning), yielding more high-quality viral sequences with less contamination (51). Despite these advances, no benchmarking has systematically compared all major viral binning tools for prokaryotic viruses. While individual publications report tool performance or limited comparisons (47, 48), none provide a direct and standardized evaluation of binning accuracy (i.e., binned viruses vs. sequenced isolated viruses), completeness, or computational efficiency across these methods.

Most downstream analysis tools accept multi-FASTA files, treating each header–sequence pair as a separate viral genome by default. As a workaround, viral contigs belonging to the same vMAG can be concatenated into a single sequence separated by stretches of ambiguous nucleotides (e.g., NNNNs), enabling their analysis as a single viral genome even with tools not specifically designed for viral bins.

All in all, binning plays a central role in generating more accurate and representative viral genome collections. While the number of tools for viral binning and their accuracy are still limited compared to those available for prokaryotes, the continuous developments and improvements will enable deeper insights into viral ecology, diversity, and evolution.

### Step 8: predicting viral lifestyle

Viral lifestyles can be described with interchangeably used terms. For clarity, we will use the terms and categories as defined by Hobbs and Abedon (53) when distinguishing between the reproduction cycle (lytic, lysogenic, and chronic) and umbrella terms (temperate and virulent). In the lytic cycle, viruses produce virions, which eventually lead to lethal host cell lysis and virion dispersal. Viruses that have only a lytic cycle are called virulent. In the lysogenic cycle, no virion production or release takes place. Instead, the virus is integrated into the host genome as a provirus and replicated along with it. Viruses able to enter the lysogenic cycle (not excluding other cycles) are called temperate. Finally, in a chronic infection, virions are produced and released over long time periods without cell lysis.

Thus, when grouping viruses into categories based on these terms, the most common categories are “lytic + temperate” (classic “proviruses” with a lytic and lysogenic cycle) and “lytic + non-temperate” (“lytic viruses” without a lysogenic cycle).

Viral lifestyle prediction is challenged by the small number of lifestyle-annotated reference genomes available, often without experimental validation (54). Instead, tools often look for different lysogenic gene markers like integrase, excisionase, recombinase, and others (55). As a result, most tools focus only on the distinction between temperate and virulent viruses.

BACPHLIP (56) assumes that all input belongs to lytic viruses and attempts to identify a lysogenic cycle based on associated genes. Since the small training data set obtained by querying the Conserved Domain Database (57) for lysogeny-associated keywords consisted almost entirely of protein domains from *Caudoviricetes* and may include proteins of different functions than annotated, BACPHLIP was trained to detect higher-level patterns in the data.

VIBRANT (23) is also able to provide the user with lifestyle predictions. It assumes all identified viruses to be lytic, except viruses excised from a host contig or viruses encoding an integrase, which are considered lysogenic.

Replidec (58) uses lysogeny-associated genes (integrase and excisionase) and is suited to process fragments <3 kb from metagenomic data. After genes are independently identified, a Naive Bayes classifier is used for probability calculation based on alignment to a custom virus database. In addition, chronic-associated genes based on the pI-like genes (morphogenesis [pI] protein) of *Inoviridae* are identified, which makes Replidec one of the few tools able to identify chronic viruses. All findings are consolidated using a voting system assuming all input sequences to belong to lytic viruses, unless lysogeny-associated genes (lytic + temperate virus) or chronic-associated genes (temperate or non-temperate chronic virus) were found.

Another common approach searches directly for sequence motifs commonly present in reference genomes of each life cycle using deep learning.

One example for this is DeePhage (59), which calculates a score for viruses identified from metagenomic data, reflecting the likelihood to be temperate or virulent based on lifestyle-specific DNA motifs.

PhaTYP (54) achieves this by building on a natural language processing model (BERT [60]) where proteins are interpreted as “words.” This will retain their biological context as part of a “sentence,” in contrast to pure k-mers and sequence motif approaches. For that, clusters of all ViralRefSeq proteins (61) were used to learn the “vocabulary” which defines general protein organization in virus genomes. Afterward, lifestyle-annotated data sets were used for fine-tuning (temperate and virulent).

The latest tool, DeepPL (55), builds on a natural language processing approach (DNABERT [62]) specifically designed for nucleotide language to overcome errors introduced through protein translation. Together with a sliding window k-mer approach, viruses are identified as temperate or virulent.

Finally, PropagAtE (63) can estimate a provirus’ infection stage. It uses assembled contigs along with the corresponding short reads or alignment information and integrated provirus coordinates. The provirus:host read coverage ratio is used to classify a provirus as active (in lytic stage of infection, significantly more provirus genomes than host), dormant (lysogenic stage), ambiguous (not active, but no evidence for dormant), or not present (provirus had no coverage). Since this tool relies on accurate sequencing results, predictions are strongly influenced by experimental biases (step 0 [Supplemental material]), and factors impacting accurate provirus coordinate predictions (step 5 [Supplemental material]).

Most available tools only distinguish between temperate and virulent viruses; however, the result remains predictive. Some of the tools achieve this by assuming all input to be from lytic viruses unless evidence for lysogenic properties is found, which can be misleading, especially in the case of lower quality viruses and non-viral input. Even though many of the mentioned tools support short virus fragments, their predictions can be influenced by potentially missing genes in fragmented viruses and wrongly included host genes of integrated proviruses. Experimental verification (e.g., plaque assays) is needed to determine the lifestyle with certainty but is often not feasible. Since all of the tools mentioned rely on annotated viral references, their performance is expected to improve as more accurate references become available.

For an overview of virus lifestyle and host interaction, see reference 64. Tool publications, including benchmarks, are provided in references 54–56, 58, 59. While performance between different tools is compared, the benchmarks are not independent. When test data overlaps with training data, the tool’s positive performance can be inflated (18).

### Step 11: viral host identification

Historically, viruses could only be identified through culture-based methods. This excludes viruses of hosts challenging to culture (e.g., from anaerobic or extreme environments), leading to culturing biases (65). Culture-independent methods mitigate this but require *in silico* host predictions. Approaches aiming to predict viral-host associations can be divided into host-based methods, which focus on association evidence within a potential host genome through host-virus comparisons, and virus-based methods, which compare the virus to viral reference genomes with a known host.

Analyzing integrated proviruses enables a straightforward host-based approach. Either the temperate virus was excised from the host, requiring host confirmation by ruling out assembly or binning errors, or the excised temperate virus (due to fragmentation or natural excision before sequencing) can be matched to an integrated provirus of a reference genome using alignment-based methods (66), with integration sites confirming matches. Viruses also tend to adapt to the nucleotide composition of the host (67), which is conserved among viruses with a similar host range and can thus be used for alignment-free host prediction by comparing k-mer frequencies, GC content, or codon usage (68). In addition, both virulent and temperate viruses can acquire host genes during infection (66, 69), so genetic homology (and thus virus-host association) can be inferred by sequence similarity searches between host and virus based on these genes. The performance of this method is dependent on the reference data and the assembly quality. However, the signal is obscured over time, as the host genome can be reorganized by other mobile genetic elements (70). Genetic evidence for more recent infections lies within bacteria and archaea containing a CRISPR/Cas system, which can be leveraged to compare viruses to a host’s CRISPR spacers. Even though not all species possess the system (71) and this method is dependent on the host references, it is highly accurate (66). Another approach considers abundance profiles, since both temperate and virulent viruses are only expected to be present together with their host. Even though the existence of host-independent states or viruses with a broad host spectrum needs to be considered, co-abundance metrics have been successfully employed to detect virus-host relationships and are especially fit to resolve interactions in time-series data sets, since their correlation can be lagged (66).

Virus-based approaches are based on the assumption that closely related viruses infect the same host genus or species (66). Such virus-virus comparisons can be made using basic sequence alignment and shared genes or nucleotide composition among viruses with a similar host range. By nature, these approaches are highly dependent on a well-curated database and less effective for novel viruses.

While some approaches can be easily implemented, there are tools that increase performance by refining and combining multiple methods. CrisprOpenDB (72) is a tool for spacer-based host prediction. It aligns user-provided viruses to a database of >11 million spacers on the nucleotide level, but it also accepts a user-provided spacer database.

SpacePHARER (73) focuses on protein-level prediction instead, since proteins are often more conserved than nucleotide sequences. Analysis can be performed starting from a virus and/or spacer, with the complementary database being provided by the tool or the user. After query and target are translated into coding sequences in six reading frames with user-definable translation tables, they are compared using MMseqs2 (39). Evidence from multiple spacer matches is reconciled.

iPHoP (74) is a pipeline for host prediction at the genus level, combining six host prediction approaches with the possibility to use the provided or a custom host reference database. Predictions of user-provided viruses using host-based methods (BLAST to host genomes to recover integrated proviruses, BLAST to a CRISPR spacer database, WIsH [75], VirHostMatcher [76], and PHP [77]) are consolidated in a host-taxonomy-aware manner and integrated with the prediction of the phage-based tool RaFAH (78). The final score is calculated, giving a higher weight to the highly precise BLAST-based predictions. Even though iPHoP also reports reference genome information on species or strain level, the developers recommend that it should only be used for genus-level predictions since it was only optimized for this purpose (S. Roux, https://bitbucket.org/srouxjgi/iphop/issues/116/how-to-choose-between).

All three host-dependent alignment-free methods focus on viruses adapting their codon usage to the host. VirHostMatcher predicts virus-host relationships employing various oligonucleotide frequency measures to calculate the distance between user-provided sequences for viruses and hosts. WIsH includes a probabilistic approach to account for k-mer frequencies becoming noisy for short viral contigs. A homogeneous Markov model is trained for each user-provided host and used to compute the prediction likelihood for each user-provided virus. PHP uses different Gaussian mixture models for varying contig lengths (including <2 kb) as a probabilistic approach instead, with the option to train customized models by providing host-virus pairs. Users can choose to compare their viruses either against a provided host database or to have their own 4-mer database built instead.

The virus-based tool RaFAH uses a machine-learning approach trained to find associations between viral proteins. Viral input is translated, and proteins are compared to HMMs of reference phage proteins with known or previously predicted hosts. Custom model training with user-provided host-virus pairs is also supported.

**Fig 4:**
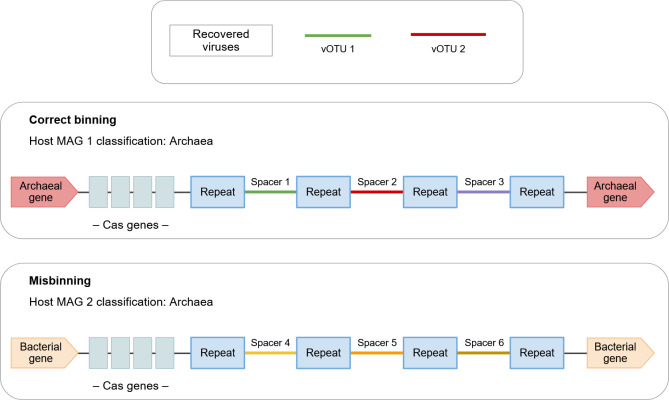
Example of a manual curation step to verify host predictions for viruses identified based on CRISPR spacer matches. Host prediction based on CRISPR spacer matches is highly accurate, as spacers are genetic evidence of recent infections. However, this is only the case if the contig containing the CRISPR array of the host is binned correctly (top). If it is misbinned (bottom), the contig—and therefore the virus-host predictions based on the spacers—will be attributed to the wrong host, leading to incorrect biological conclusions. In the example shown, a bacterial contig was misbinned into an archaeal bin. Thus, the bacterial viruses belonging to spacer 4–6 will be wrongly attributed to the archaeal host. Such errors can be mitigated by evaluating if the genes flanking the CRISPR array match the taxonomic classification of the MAG. In the case of archaea, this can be done based on matches to arCOG annotations (80).

Independent of the tool(s) chosen, the main error sources remain the same, as the prediction accuracy depends on the reference data (79). References need to be carefully curated, as host MAGs often contain contamination from misbinning events, which can lead to wrong predictions with high confidence scores. For example, spacer-based predictions of potential archaeal host MAGs can be manually curated by examining the genomic region surrounding the hosts’ CRISPR array for archaeal-specific signatures in the flanking genes (Fig. 4), such as matches to arCOG annotations (80).

Additionally, the virus data needs to be considered as an error source, since short sequences may lack significant signal to make confident predictions. Alignment-free approaches (e.g., WIsH) are more suited to handle this fragmented data than alignment-based methods (81). However, a tool’s robustness varies depending on the taxonomic level, and its accuracy generally decreases with the specificity of the prediction, which is why species-level predictions should be considered carefully (66). Results can be optimized by combining alignment-free tools most suited to handle novel viruses (high recovery and lower precision) and alignment-based tools (high precision) (82). Consolidating conflicting results can be challenging, especially when weighing the possible presence of a virus with a broad host spectrum against the performance of the tools. Furthermore, genetic markers might be indicative of different timeframes in the virus-host relationship. While spacers are evidence of recent infections, a shift in nucleotide frequencies is more indicative of an ongoing relationship (68). Experimental validation can be used to further confirm the accuracy of a prediction.

Refer to the following sources for an in-depth overview of different host prediction methods (computational and experimental) (66), application of machine learning in host prediction (83), a comprehensive list of host prediction tools, focusing also on the prediction of protein-protein interactions (84). Recent comprehensive benchmarks can be found at reference 81 (focus on virus recovery from archaeal and bacterial MAGs) and reference 85 (focus on host prediction of viruses with more than one host).

## COMPREHENSIVE PIPELINES

**Fig 5:**
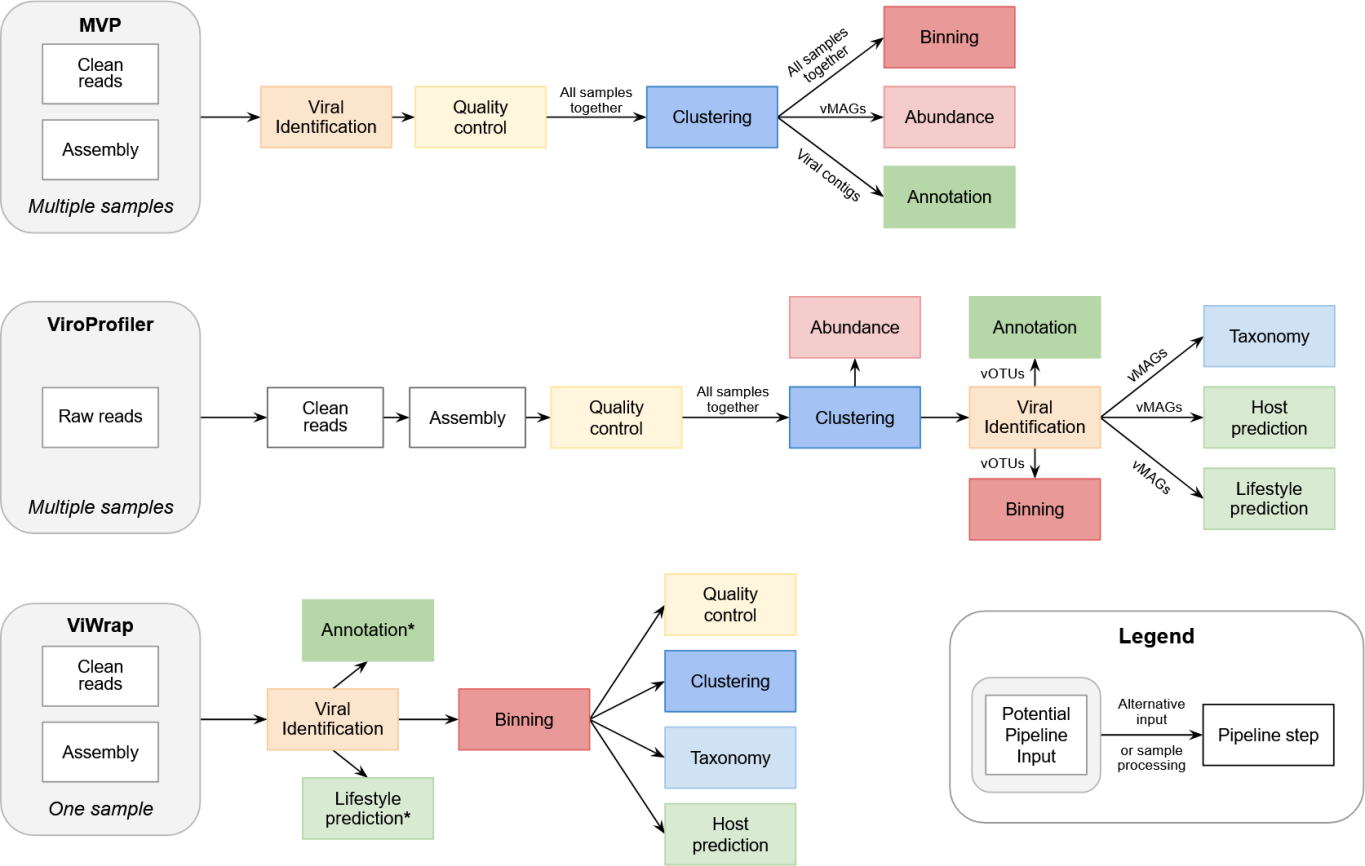
Workflow representation of the three described viromics pipelines (MVP, ViroProfiler, and ViWrap), where shared core steps are color-coded similarly. All steps are performed per sample and on the input indicated (the output from the previous tool; e.g., quality control in MVP is performed on the viral contigs). Alternative input or sample processing is noted where applicable (e.g., the abundance estimation of MVP is available for both vOTUs and vMAGs). Steps in the ViWrap pipeline depending on the viral identification tool selected are marked (*).

As the field of viral metagenomics expands, several dedicated pipelines have been developed to streamline viral genome characterization by combining multiple workflow steps for user convenience (Table 1). Among them, MVP (13), ViroProfiler (14), and ViWrap (15) are three comprehensive pipelines including a viral binning step. While all three incorporate the general workflow steps (Fig. 5), they vary in scope, modularity, and use cases. As of August 2025, no published benchmarking study has compared the three pipelines directly, although MVP and ViWrap were benchmarked against each other (13). Here, we provide a brief descriptive overview of their features to help guide potential users without evaluating their performance.

**TABLE 1:** Currently available viromics pipelines^*a*^

Parameter	MetaPhage (86)	MuDoGer (87)	MVP (13)	PhaBOX2 (88)	Sovap (89)	Veba2 (90)	VIrify (91)	ViromeFlowX (92)	ViroProfiler (14)	ViWrap (15)
Available at	github.com/MattiaPandolfoVR/MetaPhage	github.com/mdsufz/MuDoGeR	gitlab.com/ccoclet/mvp	github.com/KennthShang/PhaBOX	github.com/poursalavati/SOVAP	github.com/jolespin/veba	github.com/EBI-Metagenomics/emg-viral-pipeline	github.com/01life/ViromeFlowX	github.com/deng-lab/viroprofiler	github.com/AnantharamanLab/ViWrap
Viral identification	DeepVirFinder Phigaro VIBRANT VirFinder VirSorter2	VIBRANT VirSorter2 VirFinder	geNomad	Phamer	geNomad	VirFinder geNomad	VirSorter VirFinder PPR-Meta	VirSorter2 VirFinder	VIBRANT DeepVirFinder VirSorter2 CheckV MMseq2 taxonomy	VirSorter2 VIBRANT DeepVirFinder or geNomad
Quality/completeness	CheckV	CheckV	CheckV	Internal script	–^*b*^	CheckV	CheckV	CheckV	CheckV	CheckV
Binning	–	–	vRhyme	–	–	–	–	–	Phamb vRhyme	vRhyme
Virus clustering/dereplication	CD-HIT	gOTUpick	BLAST- based greedy clustering (provided by CheckV)	ANI or AAI	CD-HIT	FastANI	–	CD-HIT	BLAST- based clustering with scripts provided by CheckV	vConTACT2 dRep
Abundance estimation	Bowtie2 BamToCov	Kraken2 Bowtie2	Bowtie2 (short reads) Samtools Minimap (long reads) CoverM	–	Samtools	Bowtie2 Samtools SeqKit	–	Bowtie2 CoverM BEDTools	Kraken2 + Bracken Bowtie2 CoverM	CoverM
Functional annotation	DIAMOND	–	PHROGS Pfam dbAPIs RdRp HMM profiles	DIAMOND CAT/BAT PhaVIP	NCBI	UniRef50 MIBiG VFDB CAZy Pfam KOFAM	IMG/VR ViPhOG	GO EGGNOG KEGG PfamA EC CAZy	DRAM-V EggNOG ABRicate	KEGG
Taxonomy	vConTACT2	vConTACT2	geNomad	PhaGCN	geNomad	geNomad	ViPhOG NCBI taxonomy	RefSeq	vConTACT2 MMseqs2 taxonomy	RefSeq VOG vConTACT2
Lifestyle prediction	VIBRANT	VIBRANT	–	PhaTYP	–	–	–	–	Replidec BACPHLIP	VIBRANT
Host assignment	–	WIsH	–	Cherry	–	–	–	–	iPHoP	iPHoP

^*a*^ This table was already elegantly outlined by reference 13. Here, we added three additional pipelines (PhaBOX2 [88], ViroProfiler [14], and VIrify [91]) and added the lifestyle and host prediction features to the table. In the corresponding section, we describe the pipelines printed in bold. AF, alignment fraction; ANI, average nucleotide identity; AAI, average amino acid identity; HMM, hidden Markov models.

^*b*^ –, step not implemented in pipeline.

MVP is a modular workflow optimized for multi-sample studies. It uses clean reads and the corresponding assemblies from different samples as input. Viruses are predicted using geNomad (31) and assessed by CheckV (43). MVP then uses BLAST-based results and custom scripts from CheckV following MIUViG guidelines with 95% ANI and 85% alignment fraction to cluster and dereplicate identified viruses into vOTUs across all samples collectively rather than on a per-sample basis. Binning of the vOTUs is optionally performed using vRhyme (47). Abundance estimation using CoverM (93) and downstream analyses are conducted on vOTUs or vMAGs upon user selection. MVP also integrates protein prediction and annotation using the PHROGs (94) and Pfam (95) databases. Optionally, proteins are annotated using a viral anti-prokaryotic immune system protein database (dbAPIs [96]) and RNA-dependent RNA polymerase (RdRp) HMM profiles for RNA virus identification. However, host prediction and viral lifestyle classification are currently not integrated into the workflow. MVP provides structured outputs, including standardized tables and summaries, which facilitate comparative analyses across multiple conditions or environments. Its design makes it particularly suitable for researchers conducting cross-sectional or temporal studies.

ViroProfiler is a Nextflow- and container-based pipeline focusing on reproducibility through version selection. It processes raw reads to recover, annotate, and interpret viral genomes from metagenomic data. ViroProfiler begins with read preprocessing using FastQC (S. Andrews, https://www.bioinformatics.babraham.ac.uk/projects/fastqc/) and fastp (97) and assembly using metaSPAdes (98). After initial preliminary virus identification and removal of host flanking regions using CheckV, the pipeline dereplicates viral contigs across all samples to remove redundancy using BLAST results and the aforementioned custom CheckV scripts following MIUViG guidelines (same approach as MVP but different default settings). For abundance estimation, Kraken2 (99) with a custom ViralRefSeq database and Bracken (100) provide read-based taxonomic profiles for known viruses. Additionally, reads are mapped to assembled preliminary viral contigs using Bowtie2 (101), with coverage filtered and calculated by CoverM to estimate viral abundance including novel viruses. The following viral identification uses a combination of results from CheckV, MMseqs2 (39), taxonomy assignment based on ViralRefSeq (61), VirSorter2 (26), DeepVirFinder (27), and VIBRANT (23), while genome quality is assessed through CheckV. Binning can be optionally performed with vRhyme (viral contigs) or phamb (102) on dereplicated preliminary viral contigs. Taxonomic classification of assembled viral genomes is achieved through genus-level clustering by vConTACT2 (42), and protein searches against ViralRefSeq using MMseqs2. Host prediction is performed using iPHoP based on its default reference database. Functional annotation and AMG prediction are conducted primarily with DRAM-v, supplemented by eggNOG-mapper (103) and ABRicate (T. Seemann, https://github.com/tseemann/abricate). Lifestyle prediction tools BACPHLIP (56) and Replidec (58) are included as optional steps.

ViWrap is a modular pipeline optimized for single sample studies run on assemblies with the option to add clean reads. It also provides the option to add MAGs from the same sample for targeted host prediction. ViWrap performs viral identification using different tool combinations depending on the version and user settings. The current version allows the use of DeepVirFinder, VirSorter2, and VIBRANT either independently or by consolidating their outputs. If geNomad is selected instead, downstream steps are applied only to geNomad-predicted viral contigs. VIBRANT is required if contigs should be annotated, since it performs general gene prediction and annotation using Prodigal. However, VIBRANT also enables lifestyle prediction by annotating viral genomes with indicators of lytic or lysogenic potential and prediction of AMGs. Binning is a required step conducted using vRhyme, and CheckV is used to assess quality only after binning, not before. CheckV runs on each vMAG separately, producing one output folder per vMAG, reducing computational load for large data sets. Dereplication is then performed using dRep (36) to generate vOTUs out of vMAGs as input. All analyses, including dereplication and binning, are performed on a per-sample basis, rather than across samples. Viral taxonomy is inferred through a combination of ViralRefSeq protein searches, HMM-based marker detection from the VOG database (104), and vConTACT2 clustering, which incorporates genus-level clusters anchored by high-quality vOTU representatives from IMG/VR v4 (105). Host prediction is performed using iPHoP (74), either with the default reference database or by supplementing it with the user-provided MAGs. ViWrap is especially suitable for researchers interested in per-sample viral community structure, virus-host interactions, and functional diversity.

These pipelines, despite overlapping components, can all be applied to diverse viral metagenomics data sets. The choice depends on experimental design and how users wish to organize and present their data. This includes preferences toward tools used for individual steps (Table 1), the order in which the steps are performed, and whether the intended focus is viral contigs, vMAGs, or vOTUs (Fig. 5).

MVP is a compact pipeline to generate the base data for further virome analyses from multiple samples based on assembled contigs. It also includes the prediction of viral anti-prokaryotic immune systems and RNA viruses. It follows a robust workflow focused on information retention by prioritizing viral identification based on the whole data set, rather than reducing the computational workload early on. As a result, it generates vOTUs from viral contigs prior to the optional viral binning step. While the focus is on vOTUs and data are dereplicated across samples, alternative inputs can be specified. Additionally, MVP prepares input files needed for manual execution of DRAM-v (AMG prediction) and iPHoP (host prediction). However, neither these tools nor a lifestyle prediction step is included in the pipeline. MVP’s strength lies in its well-organized and structured output. Files from any step can be easily located and used in user-specific downstream analyses based on non-included tools.

ViroProfiler is particularly suited to generate a quick viral overview of multiple samples based on raw data, as it includes read preprocessing, assembly, and early data reduction by dereplication across all samples. Since CheckV is employed prior to exhaustive viral detection, this pipeline is best used with enriched samples. Downstream analyses can be performed flexibly on viral contigs or optionally on vMAGs and include prediction of host, lifestyle, and AMGs.

ViWrap is a vMAG-focused workflow for single-sample analyses based on assembled contigs. Binning is required, and most steps are performed on vMAGs (including clustering and viral quality assessment), which makes ViWrap particularly suitable for fragmented assemblies. Annotation and lifestyle prediction require the use of VIBRANT but can be added manually later on by using any suitable tool. A benefit of this pipeline is the support of custom MAGs for host prediction.

Users should choose a pipeline based on their data set structure and needs, research questions, and desired analyses, consulting documentation for current capabilities and potentially added features in future tool updates. Any missing functionality can always be supplemented manually through other tools based on user needs, while future benchmarking studies will help clarify comparative performance.

## CONCLUSION AND OUTLOOK

In this work, we presented an overview of all steps necessary for a virome analysis that serves as a valuable resource to newcomers and more experienced users alike. While integrating each step from data acquisition and preprocessing over viral identification and viral quality control to clustering, binning, and common downstream analyses, we provide users with methodological background. The supplemental material offers details on all the described tools, so users can easily select and combine what fits their analysis best. Additionally, we provide an overview of the most common issues that can arise during any of the described steps and suggest troubleshooting approaches. Together, this guide helps users make informed and confident decisions about which steps their data requires and how to answer their research questions.

The emergence of new technologies and methods will further advance how viromics is approached. Artificial intelligence-based applications are already on the rise and used in, e.g., viral identification, binning, lifestyle prediction, and host identification as mentioned throughout the manuscript. In addition, structural prediction has advanced rapidly in the past few years. Due to the ever-improving speed and accuracy of prediction methods and the availability of high-performance computing, structural prediction can now be applied to large data sets (106). The resulting databases of viral protein structures (e.g., Viro3D [107]) are not only beneficial for structure-guided phylogeny (108) and annotation (109) but can also be used for viral identification. This was already applied to identify potential RNA viruses based on potential RdRp sequences by combining insights from sequence data and structural predictions (LucaProt [110]). In the future, these developments will aid in the identification of highly divergent viruses that remain uncharacterized to date. While these developments are powerful if applied correctly, experimental validation will remain a cornerstone of viral characterization.

## SUPPLEMENTAL MATERIAL

Supplemental material msystems.01249-25-s0001.docx Supplemental table captions and text; Table S4.

Supplemental tables msystems.01249-25-s0002.xlsx Tables S1–S3.

ASM does not own the copyrights to Supplemental Material that may be linked to, or accessed through, an article. The authors have granted ASM a non-exclusive, world-wide license to publish the Supplemental Material files. Please contact the corresponding author directly for reuse.
