# Supplementary material for: Navigating prokaryotic viral genome analysis from metagenomic data
Source: mSystems. 2026 Apr 21;11(5):e01249-25. doi: 10.1128/msystems.01249-25 (PMC13185550; doi:10.1128/msystems.01249-25)
Supplement: Supplemental material — Supplemental table captions and text; Table S4. [file msystems.01249-25-s0001.docx]

## Supplementary Material

#### **Table S1: Overview of tools for viromic analyses and their capabilities**

Described are all major tools mentioned in the manuscript and supplementary material, if they have a virus-specific mode or function (including the option to select a virus-specific database), are compatible with metagenome-derived data, include a functionality specific to integrated proviruses (e.g. host-virus border awareness), are optimized to handle also short or novel viruses, and support custom database selection or model training outside of their default functionality. Specifications not applicable for a tool are marked with a slash. Additionally, we included short descriptions, important specifics, or limitations to aid users in selecting the tools best suited for their specific goals.

#### **Table S2: Additional resources for viromic analyses**

This is a comprehensive list of additional tools not described in the manuscript.

#### **Table S3: Troubleshooting common issues arising during viromic analyses**

Described are behaviors and problematic results commonly arising during any of the described workflow steps. We list potential causes and proposed next steps sorted by suggested order of operation. Potential next steps are not mutually exclusive.

### **Step 0: Experimental considerations**

Before the steps necessary for virome analysis are explored, it should be noted that common biases may already arise during sample preparation. These biases should be considered when planning the experiment and analysis to suit specific needs. Variations in sample type, sample source, and virus may lead to different effects; so the biases to be accounted for will depend on the intended goals and questions.

A sample is either collected directly from a host or host community (**non-enriched sample**), or it is enriched for viruses prior to sample processing (e.g. by filtration) with the possibility to sequence either the whole viral metagenome, or to select single virus genomes (**enriched sample**) (Table S4). A shared trait is that the recovered viruses will always only represent a subset of the true community composition [(1)](https://paperpile.com/c/rUn8Q0/MI85). This is due to the nature of randomly sampling a community, and also to the different shapes, sizes and physical properties of viral community members, thus resulting in different responses to the stress occurring during sample processing [(2)](https://paperpile.com/c/rUn8Q0/hbo1). Therefore, a protocol’s efficiency (both in yield of nucleic acids and recovery of viral signals) can vary depending on the sample source [(3)](https://paperpile.com/c/rUn8Q0/SWy1). This is influenced by the source-specific community composition and viral density within the sample, and also potentially by the sample's own properties (e.g. pH, salinity, or presence of particles or debris). Even the type of buffer used to resuspend a sample can impact the results, since different ions can make viruses more susceptible to inactivation at high temperature (60°C) (detailed in [(4, 5)](https://paperpile.com/c/rUn8Q0/Uhnp+YI6D)). This is especially important to consider when comparing virome variability or diversity, and in particular when comparing data from multiple sources obtained through different protocols.

Often, samples are enriched for virus-like particles (VLPs) by physically separating the viral fraction using small-pored filters. This enrichment aims to remove background noise from other members of the community or any genetic contamination possibly introduced during handling. This background noise tends to complicate viral identification by obscuring the viral signal due to the difference in sequence depths [(1)](https://paperpile.com/c/rUn8Q0/MI85). Virus particle size can range from 20 nm in diameter (*Picornaviridae*) up to 400 nm (*Mimivirus, Megavirus*), with exceptional species forming elliptical virions of up to 1 µm in length and 700 nm in width (*Pandoravirus*) or filamentous shapes of over 1 µm in length with a diameter of about 80 nm (*Filoviridae*) [(6)](https://paperpile.com/c/rUn8Q0/31K9). Thus, the commonly used 0.22 µm filters will not only exclude microbial cells, but also larger viral particles or particles with a filamentous shape. This can lead to a reduction of recovered viral particles by up to 50% in comparison to using 0.45 µm filters [(7)](https://paperpile.com/c/rUn8Q0/jzRH). Even though no bacterial contamination was previously observed when using 0.45 µm filters [(7, 8)](https://paperpile.com/c/rUn8Q0/jzRH+PEUJ), small bacteria like *Mycoplasma* with a diameter of 200-300 nm [(6)](https://paperpile.com/c/rUn8Q0/31K9), bacterial endospores with a diameter of 0.25 µm [(9)](https://paperpile.com/c/rUn8Q0/CMwI), or vesicles containing host material [(10)](https://paperpile.com/c/rUn8Q0/OMet) can potentially also pass through those filters. Additionally, host DNA contamination in enriched samples can come from the virus particles themselves, when genetic material of non-viral origin was packaged into the capsids (generalised and specialised transduction) [(11)](https://paperpile.com/c/rUn8Q0/8iPb). There are many different filter shapes, materials, and setups available that each have a unique effect on viral recovery due to their morphology, charge and hydrophobicity [(8, 12, 13)](https://paperpile.com/c/rUn8Q0/PEUJ+oGET+Qtmz).

Another common step to concentrate samples is polyethylene glycol (PEG) precipitation. Even though it includes an overnight incubation step, it performs well regarding cost/efficiency ratio and reproducibility in comparison to other methods, making it a good fit for large-scale studies [(14)](https://paperpile.com/c/rUn8Q0/tqWW). However, if chloroform is used to remove PEG (alternatives: [(15)](https://paperpile.com/c/rUn8Q0/6vSd)), it can degrade the phospholipid membrane of some enveloped viruses (e.g. dsRNA viruses of the family *Cystoviridae*), thus leading to their underrepresentation [(5)](https://paperpile.com/c/rUn8Q0/YI6D). Other approaches like flow cytometry-based methods aim to separate VLPs from the background noise by labelling the viruses with a fluorescent dye before selecting them based on size and fluorescence level followed by an extraction using fluorescence-activated cell sorting. Even though this method decreases the amount of recovered viruses, it can eliminate the need for amplification steps before sequencing [(16)](https://paperpile.com/c/rUn8Q0/Ry2W).

Many samples with small volume or low viral concentration (e.g. skin swabs or seawater) call for amplification steps prior to sequencing which can enrich for or select against certain viruses. For example, even though multiple displacement amplification (MDA) is intended to amplify DNA non-specifically, it is biased towards preferentially amplifying small and circular ssDNA, decreasing diversity and leading to less reproducible results [(5)](https://paperpile.com/c/rUn8Q0/YI6D). MDA also drastically reduces the abundance of dsDNA phages to the point of nearly excluding them and thus should be avoided when focusing on dsDNA phages [(14)](https://paperpile.com/c/rUn8Q0/tqWW). This amplification method was additionally found to unevenly amplify linear genome fragments [(17)](https://paperpile.com/c/rUn8Q0/r9k3) and is unable to amplify RNA viruses [(5)](https://paperpile.com/c/rUn8Q0/YI6D). Other library methods like linker amplification or tagmentation select against ssDNA viruses, therefore efforts were made to quantify these biases, develop a protocol able to accurately represent both dsDNA and ssDNA viruses, and to determine the true abundances of both [(17)](https://paperpile.com/c/rUn8Q0/r9k3).

Upon comparison, the best performing bioinformatic tool combination identified a higher proportion of viral sequences in the virus-enriched samples (44%-46%) than in non-enriched metagenomes (7%-19%) (aquatic samples, [(18)](https://paperpile.com/c/rUn8Q0/CEs6); individual proportions will differ between environments [(3)](https://paperpile.com/c/rUn8Q0/SWy1) but the discrepancy remains). Nevertheless, it is important to employ both enriched and non-enriched samples to capture the full virosphere while also being mindful of their unique challenges [(19)](https://paperpile.com/c/rUn8Q0/F2Nb).

Refer to the following sources for an overview of the most common sample preparation biases [(16)](https://paperpile.com/c/rUn8Q0/Ry2W), viral families and which substances commonly used in protocols their virions are sensitive towards as well as a more detailed discussion of different protocols [(3)](https://paperpile.com/c/rUn8Q0/SWy1), common pitfalls during extractions for RNA viruses [(5)](https://paperpile.com/c/rUn8Q0/YI6D), and the effect of different storage conditions on bacteriophages [(20)](https://paperpile.com/c/rUn8Q0/MxOS).

#### **Table S4: Non-enriched and virus-enriched samples, their applications and common issues**

| **Sample type** | **Details** |
| --- | --- |
| **General** | - Certain chemicals as well as filtration and amplification steps introduce significant biases into virome abundance and composition by enriching for or selecting against certain viruses [(5)](https://paperpile.com/c/rUn8Q0/YI6D) - Protocol efficiency can vary depending on the sample source [(3)](https://paperpile.com/c/rUn8Q0/SWy1) |
| **Non-enriched samples** | - Either from single host genome (e.g. from cultivated cells or single-cell sorting) or host community metagenome (e.g. environmental or clinical sample) - Will maintain information about community composition and abundances - Enables research of both virus and host characteristics - Virus-host association is maintained with high fidelity (especially in genomic samples) - High background noise from host or metagenome complicates identification of viral signals [(1)](https://paperpile.com/c/rUn8Q0/MI85) - Can capture: host-associated viruses (attached to the hosts’ surface, intracellular or integrated into the host genome) and free viruses - Over-representation: Virulent viruses with high burst size (number of viral particles released during lysis of infected cell), viruses infecting the dominant host [(19)](https://paperpile.com/c/rUn8Q0/F2Nb) - Under-representation: Rare viruses or viruses infecting rare hosts [(19)](https://paperpile.com/c/rUn8Q0/F2Nb) |
| **Enriched samples** | - Either from single viral genome (single particle separation through e.g. flow cytometry) or viral metagenome (concentration of separated particles through physical or chemical means) - Increased confidence that the sequence is of viral origin - Less background noise from non-viral sequences, but information loss due to the selection process [(5)](https://paperpile.com/c/rUn8Q0/YI6D) - Low background noise benefits *de novo* assembly, identification of low abundance viruses, and identification of individual genotypes in mixed populations - Potential to specifically target specific viruses, e.g. in clinical sample, or to optimize protocol for expected viral species - Host range only through *in silico* prediction - Can capture: Free viruses (including non-integrated proviruses and extracellular vesicles containing viral genetic material [(10)](https://paperpile.com/c/rUn8Q0/OMet)) - Can capture at lower efficiency: Viruses associated with the cellular fraction (attached to cell surface or intracellular), but only if host cells are disrupted - Can not capture: Viruses integrated into host genome - Over-representation: Virulent viruses with high burst size [(19)](https://paperpile.com/c/rUn8Q0/F2Nb) |

### **Step 1: Data preprocessing**

While sequencing techniques also influence viral recovery, they have been discussed in detail elsewhere [(21, 22)](https://paperpile.com/c/rUn8Q0/QpD8+DzOo) and thus are not the focus of our guide.
A typical metagenomics workflow starts with quality assessment of the obtained reads to identify and remove low-quality sequences, adapter contamination, and other technical artifacts that could affect downstream analyses. Tools like **FastQC** (Babraham Institute, <https://www.bioinformatics.babraham.ac.uk/projects/fastqc/>) or **fastp** [(23)](https://paperpile.com/c/rUn8Q0/16qp) quantify sequence quality per base, GC content, sequence length distribution, sequence duplication levels, and other metrics to enable the user to filter using default parameters or their own selection criteria. Some of the QC tools like **fastp** trim adapters automatically. But there are also a multitude of tools specifically for that purpose, e.g. **CutAdapt** [(24)](https://paperpile.com/c/rUn8Q0/7Q7v) or **trimmomatic** [(25)](https://paperpile.com/c/rUn8Q0/WfpW). Vectors can be removed by comparing the reads with the **UniVec database** (<https://www.ncbi.nlm.nih.gov/tools/vecscreen/univec/>) (using e.g. **BLAST** [(26)](https://paperpile.com/c/rUn8Q0/Gs31) or **Kraken2** [(27)](https://paperpile.com/c/rUn8Q0/yuxt))**,** which contains vector, adapter, linker, and primer sequences, while other specialized databases (Table S1) can be used to filter out contaminations from hosts or unwanted organelles. It is advisable to re-estimate read quality afterwards and evaluate the need for additional trimming or filtering.

Refer to the following sources for additional information (J. Blanca & J. Cañizares, <https://bioinf.comav.upv.es/courses/sequence_analysis/read_cleaning.html>), as well as examples of good and bad quality reports for different sequencers (Babraham Institute, <http://www.bioinformatics.babraham.ac.uk/projects/fastqc/>).

### **Step 2: Read profiling**

A read profiling step can be performed on the clean reads to generate a quick taxonomic overview of the sample(s). This step is not commonly included in ready-to-use pipelines. However, since not all reads will be assembled into contigs, and not all contigs will end up in downstream analysis, it can be beneficial to get an unfiltered overview of the data (Fig. 2). **Kraken2** [(27)](https://paperpile.com/c/rUn8Q0/yuxt) compares k-mers of the reads with available precompiled or custom databases (supports genome, protein and 16S sequences) and assigns the appropriate taxon for each sequence, while **Bracken** [(28)](https://paperpile.com/c/rUn8Q0/Yl03) estimates the abundance per taxon of the resulting output. **Phanta** [(29)](https://paperpile.com/c/rUn8Q0/a62V) is a workflow optimized for gut viromes that combines **Kraken2,** **Bracken,** and gut-specific databases. It offers optional post-processing scripts like collapsing viral abundances in each sample by the predicted host. There are also tools that use a non-k-mer-based approach. **Kaiju** [(30)](https://paperpile.com/c/rUn8Q0/TgEB) (only supports protein databases) uses a backwards search algorithm based on a modified data compression algorithm (Burrows-Wheeler transform [(31)](https://paperpile.com/c/rUn8Q0/fFAd), used in compression tools like bzip2 (J. Seward, <https://sourceware.org/bzip2/>) or mapping tools like Bowtie2 [(32)](https://paperpile.com/c/rUn8Q0/6A1o) or BWA [(33)](https://paperpile.com/c/rUn8Q0/pFaP)), while **MetaPhlAn4** [(34)](https://paperpile.com/c/rUn8Q0/rAA9) maps reads against a curated database of marker genes using Bowtie2.

Additionally, read profiling can help capture issues caused by sample preparation or sequencing early on (see Table S3); e.g. by uncovering host contamination. Host contamination in enriched samples specifically can also be estimated using **ViromeQC** [(35)](https://paperpile.com/c/rUn8Q0/MUVd). This tool maps reads against a reference database of rRNAs and single-copy bacterial markers. It then calculates a viral enrichment score by comparing the abundance of these markers to medians observed in non-enriched reference metagenomes (human, environmental or custom). While this tool has not been updated recently, no alternative exists to our knowledge.

For a benchmark of the mentioned and additional taxonomic classifiers, refer to [(36)](https://paperpile.com/c/rUn8Q0/QzSt).

### **Step 3: Assembly**

Assembly aims to connect reads into larger contigs. Assembly tools or pipelines are often optimized for specific read lengths (short or long), sample types, or domains. The assembly of metagenomic samples is especially challenging, as they frequently contain an uneven abundance of organisms found in a sample, genomic variation between closely related organisms (microdiversity), conserved genomic regions shared by distantly related genomes (inter-genomic repeats) and repetitive DNA or insertion elements within a single genome (intra-genomic repeats), which is a general issue for assemblers [(37)](https://paperpile.com/c/rUn8Q0/6Urc). Popular tools suitable for viral assembly from enriched and non-enriched (meta-)genomic samples include **meta(viral)SPAdes** [(38, 39)](https://paperpile.com/c/rUn8Q0/IJCG+CAs9) and **MEGAHIT** [(40)](https://paperpile.com/c/rUn8Q0/DIks). Often, the reconstruction of longer contigs is achieved at the expense of microdiversity. Variant-aware scaffolding tools like **MetaCarvel** [(37)](https://paperpile.com/c/rUn8Q0/6Urc) aim to regain microdiversity information by using read mapping to connect contigs into longer scaffolds.

An option to overcome the issues of repeat regions within viral genomes is hybrid assemblies. Short reads often fail to span an entire repeat region, resulting in generally more fragmented assemblies. While long reads span larger regions they have higher error rates than short reads [(41)](https://paperpile.com/c/rUn8Q0/zqw9), although the error rates have been steadily declining since the introduction of long-read sequencing [(22)](https://paperpile.com/c/rUn8Q0/DzOo). A hybrid assembly combines the strengths of both short and long read types by first creating an assembly using only one type, and then using the other to polish the assembly. A comparison by Wick et al. [(41)](https://paperpile.com/c/rUn8Q0/zqw9) found that the long-read-first hybrid assembly approaches outperformed the short-read-first assemblies. It is important to note that resequencing a stored sample to obtain the other read type for hybrid assembly can cause misleading results. Even though storage temperature and repeated freezing have been found to only mildly affect the virome [(42)](https://paperpile.com/c/rUn8Q0/crXD), they can impact the hosts in metagenomic samples [(43)](https://paperpile.com/c/rUn8Q0/vl1u). Additionally, the genetic information can slightly change during the storage time simply because biological processes can still take place [(44)](https://paperpile.com/c/rUn8Q0/NIh1). Therefore, it is preferable to perform both sequencing types for the same sample (and optimally DNA preparation) in close temporal proximity when intended to be combined during analysis.

Refer to the following sources for details about the assembly of bacterial isolates and the general workflow of hybrid assemblies (R. Wick, <https://rrwick.github.io/2020/10/30/guide-to-bacterial-genome-assembly.html>), a benchmark of different sequencing and assembling protocols [(22)](https://paperpile.com/c/rUn8Q0/DzOo), and a benchmark of different metagenomic assembly tools with a special focus on virome recovery [(45)](https://paperpile.com/c/rUn8Q0/4n57).

### **Step 5: Viral quality assessment**

Viral quality is typically assessed regarding completeness and contamination. Completeness describes how much of a virus is recovered. This requires prior knowledge of what kind of virus to expect. Either, there is an exact reference for the virus available, or completeness is estimated based on the length of close relatives (applicable for both free and integrated viruses; circular and linear). For example, **viralComplete** [(39)](https://paperpile.com/c/rUn8Q0/CAs9) estimates completeness based on the assumptions that virus genome size is consistent across a viral family, and that a novel virus will have a similar gene content compared to viruses of the same family. A different approach is based on the automated detection of circularization signatures like terminal nucleotide repeats (direct or inverted) (e.g. **VIBRANT** [(46)](https://paperpile.com/c/rUn8Q0/GCgL) or **CheckV** [(47)](https://paperpile.com/c/rUn8Q0/vJ0m). While it is a good indicator for the completeness of circular free viruses, it leads to complete linear genomes frequently being identified as high quality or near complete, since they are often lacking those repeats [(1)](https://paperpile.com/c/rUn8Q0/MI85). Consequently, linear genomes tend to be disregarded when searching for (complete) reference genomes. It can also lead to incomplete viral genomes containing repeats being incorrectly classified as complete circular genomes [(48)](https://paperpile.com/c/rUn8Q0/OFkH), thus, tools often include additional approaches.

Contamination describes how much of a sequence is non-viral. It can stem from genes acquired during generalised or specialised transduction [(11)](https://paperpile.com/c/rUn8Q0/8iPb), from genes and AMGs introduced by transposable elements [(49)](https://paperpile.com/c/rUn8Q0/go2P), prediction errors, or misassemblies [(50)](https://paperpile.com/c/rUn8Q0/D1Ei). Contamination and completeness estimation of integrated proviruses both require accurate prediction of the border between viral and host genes. This poses a challenge, since viral genomes can contain genes common to both viruses and host [(1)](https://paperpile.com/c/rUn8Q0/MI85), and since potential host references from public databases can be contaminated with undeclared integrated proviruses [(18)](https://paperpile.com/c/rUn8Q0/CEs6). Wrongly predicting these borders leads to either remaining host contamination, or loss of information due to excessive trimming. This can affect downstream analysis, especially the correct estimation of viral genome size (e.g. completeness estimations) and characterization of the viral metabolic potential [(47)](https://paperpile.com/c/rUn8Q0/vJ0m).

Many viral identification tools provide a confidence score for viral origin. **VIBRANT** uses viral hallmark genes, circularization signatures, and other metrics to classify sequences as complete circular, high-quality draft, medium-quality draft, or low-quality draft, and assigns a v-score to protein annotations to reflect similarity to viral genomes, though not distinguishing overlap with host genes. Sliding window analysis of v-score also helps detect integrated proviruses [(46)](https://paperpile.com/c/rUn8Q0/GCgL). **VirSorter2** combines features such as hallmark genes, gene size, and strand switching to score sequences from 0 to 1, with values above 0.9 indicating high confidence and below 0.5 low confidence [(51)](https://paperpile.com/c/rUn8Q0/cWAE). **ViralVerify** applies a naive Bayes classifier to label sequences as Chromosome, Plasmid, Virus, Unclassified, or Uncertain, while also providing likelihoods for each option [(39)](https://paperpile.com/c/rUn8Q0/CAs9).

**CheckV** is a tool developed for viral quality assessment. It uses circularization signatures and reference comparisons to evaluate completeness and to extract proviruses. Host-virus boundaries are detected with a sliding window that combines GC content differences and annotations from archaea, bacteria, and virus-specific HMMs. Genome completeness is estimated using the best hit based on average amino acid identity (AAI), where alignment strength against a custom database of complete viral genomes is converted into a confidence level. If no close match is found, completeness is inferred from virus-specific HMMs and the length distribution of reference genomes matching the same viral HMMs. Closed genomes are predicted using direct or indirect terminal repeats as circularization signatures, or host boundaries at both ends for integrated viruses, taking completeness into account. Sequences are then classified as Complete, High-quality (>90% completeness), Medium-quality (50–90%), Low-quality (<50%), or Undetermined.

While **CheckV** is widely used for viral quality assessment, it has clear limitations. Built for quality evaluation rather than viral identification, non-viral input may be reported with 100% completeness and 0% contamination. This occurs because the tool assumes input is viral and searches for a non-existing host-virus border, thus reporting 0% contamination. Completeness is then estimated from “viral” genes also found in non-viral sequences; sometimes using a shorter viral representative than the non-viral input to infer genome size. As a result, sequences can appear 100% complete and High-quality, since quality categories rely only on completeness as host contamination is trimmable. Such results can mislead inexperienced users, so **CheckV** should be applied only after viral identification. Its gene and genome detection also depends on custom databases that do not represent all viruses and hosts equally. Nevertheless, **CheckV** remains the standard tool for viral quality estimation and is integrated into public viral databases workflows (e.g. IMG/VR, [(52)](https://paperpile.com/c/rUn8Q0/G5kS)).

Refer to [(47)](https://paperpile.com/c/rUn8Q0/vJ0m) for a benchmark of different tools used for viral quality assessment.

### **Step 9: Viral taxonomy**

Viral taxonomy is governed by the ICTV, which defines official standards for virus naming and classification [(53)](https://paperpile.com/c/rUn8Q0/vEbJ). Its system is hierarchical, with required genus and species assignment, and optional use of higher ranks. Historically, classification relied on features like morphology, genome type, and host range, however is now primarily genomics-driven. To accommodate the immense viral diversity, the ICTV adopted a megataxonomy framework [(54)](https://paperpile.com/c/rUn8Q0/1TGl) that adds ranks above family and order, including realm, kingdom, phylum, and class. Despite the polyphyletic origins of viruses [(55)](https://paperpile.com/c/rUn8Q0/e81e) and lack of universal viral genes, realms are organized by conserved hallmark genes. It is especially valuable for integrating novel viral lineages, advancing understanding of viral evolution and diversity.

Building on this framework, viral taxonomic classification relies on sequence type and rank. Higher levels (order or family), use conserved protein alignments that remain detectable over long evolutionary distances [(56, 57)](https://paperpile.com/c/rUn8Q0/vnta+3tv6). In contrast, genus- and species-level assignments commonly use nucleotide sequence identity and whole-genome alignments, providing finer resolution for closely related viruses. This level-based approach enables robust and standardized classification across the full spectrum of viral diversity.

To implement these principles, a variety of computational tools with distinct strategies tailored to data type and classification goals were developed. One prominent strategy leverages gene-sharing networks. **vConTACT2** [(58)](https://paperpile.com/c/rUn8Q0/3rEg) clusters viral genomes by shared gene content, allowing tentative genus-level or higher taxonomy assignments. This approach is robust for incomplete or highly divergent genomes and useful for discovering novel viruses.

Another method are phylogeny-aware tools which explicitly construct trees based on evolutionary distances. **ViPTree** [(59)](https://paperpile.com/c/rUn8Q0/e1ek) constructs viral phylogenetic trees using the whole proteome, enabling visualization of evolutionary relationships particularly at higher taxonomic levels. **VICTOR** [(60)](https://paperpile.com/c/rUn8Q0/kc6p) computes genome-to-genome distances and infers phylogenetic trees supporting robust classification at species, genus, subfamily, and family levels. **GRAViTy-V2** [(61)](https://paperpile.com/c/rUn8Q0/9tUu) uses alignment-free distance calculations derived from profile HMMs and genomic organization models. It produces taxonomies consistent with ICTV down to family and often to genus and species levels. **VIRIDIC** [(62)](https://paperpile.com/c/rUn8Q0/cGNY) focuses specifically on calculating pairwise intergenomic similarities to cluster viruses at genus and species demarcation levels.

Protein clustering and core gene detection represent another strategy. **VirClust** [(63)](https://paperpile.com/c/rUn8Q0/eUfy) applies sensitive homology searches followed by HMM comparisons to define protein clusters and hierarchically cluster genomes. It identifies core proteins and viral hallmark genes to assign taxonomy at multiple levels.

**GeNomad** [(64)](https://paperpile.com/c/rUn8Q0/18Qt) applies a hybrid approach combining marker gene annotation with similarity-based and machine learning methods enabling classification of divergent and incomplete genomes. It assigns taxonomy by aligning predicted genes to a comprehensive set of viral hallmark genes, aggregating results into a consensus lineage.

Sequence similarity based taxonomy assignment is also widely used. **MMseqs2** (taxonomy workflow, [(65)](https://paperpile.com/c/rUn8Q0/y4mN)) identifies significant matches to reference sequences, then assigns the most specific taxonomic rank shared by all high-confidence hits. This method ensures conservative and accurate taxonomic classification, especially when top hits span multiple related taxa.

Lastly, a combined alignment-based and graph-based approach is implemented by **VITAP** [(66)](https://paperpile.com/c/rUn8Q0/Pfg1), which was evaluated for both DNA and RNA viruses at family and genus levels across diverse datasets. VITAP demonstrates improved annotation rates particularly for short or incomplete contigs.

In general, tools benchmark their taxonomic assignment against curated ICTV assignments. The results generally correlate with the structural, biological, and morphological characteristics of ICTV viruses. Most tools are challenged by incomplete genome sequences, the absence of hallmark genes needed for resolution of novel families and genera, and their performance is constrained by the quality and completeness of reference databases (relevant benchmarks: [(58, 64, 66, 67)](https://paperpile.com/c/rUn8Q0/3rEg+18Qt+AE7K+Pfg1)). Therefore, sequence-driven taxonomic assignments should be considered provisional, as short or highly divergent contigs often lack sufficient signal for confident classification. It is essential to interpret results cautiously and, whenever possible, support taxonomic claims with complementary evidence or experimental validation.

An emerging outlook applied in several recent studies [(54, 68, 69)](https://paperpile.com/c/rUn8Q0/UlYQ+1TGl+snFw) is structure-guided taxonomy, where predicted three-dimensional structures of viral (hallmark) proteins are compared using their underlying evolutionary relationship for taxonomic classification. A promising stand-alone pipeline is **Unicore** [(70)](https://paperpile.com/c/rUn8Q0/T4pC), which generates a structure-guided phylogenetic tree of all input proteomes putting them into context. Although no formal taxonomic classification is performed, it can be achieved by including input with known taxonomy. Thus, structure-guided classification based on protein fold comparisons is advancing as a complement to sequence-based taxonomy, though not yet fully automated.

Refer to the following sources for details about experimental and computational classification methods [(71)](https://paperpile.com/c/rUn8Q0/FLXd), taxonomy [(72)](https://paperpile.com/c/rUn8Q0/35N4) and establishing taxonomy [(73)](https://paperpile.com/c/rUn8Q0/rFkh), virus nomenclature [(74)](https://paperpile.com/c/rUn8Q0/fcD3), and taxonomic classification of uncultivated viruses [(75)](https://paperpile.com/c/rUn8Q0/FE4T).

### **Step 10: Viral abundance estimation**

Viral abundance estimation quantifies how much each viral genome is represented in a sample, providing insight into the population dynamics and community structure of viruses. Reads from each sample are mapped to the dereplicated vOTU representatives using aligners like **Bowtie2** [(32)](https://paperpile.com/c/rUn8Q0/6A1o) or **BWA** [(33)](https://paperpile.com/c/rUn8Q0/pFaP), allowing for quantification of novel viruses that may not be present in reference databases. Tools such as **CoverM** [(76)](https://paperpile.com/c/rUn8Q0/hpCE) can process these alignments to generate per-genome statistics, including raw and normalized read counts, mean coverage, and fraction of the genome covered. Normalization of mapped read counts is essential for comparing viral abundances across samples. Although CoverM was not specifically developed for estimating viral abundance, it performs robustly for this purpose.

Alternatively, classification-based approaches such as **Kraken2** [(27)](https://paperpile.com/c/rUn8Q0/yuxt) can assign reads to known viral taxa, with **Bracken** [(28)](https://paperpile.com/c/rUn8Q0/Yl03) re-estimating species- or genus-level abundances from these assignments. However, these methods are dependent on the reference genomes present in the database and do not account for genome length, making them suitable only for viruses closely related to known references.

It is important to note that certain amplification protocols prior to sequencing can introduce compositional biases (Step 0, Supplementary). The MIUViG guidelines advise that abundance estimates from such amplified viromes can indicate presence or absence of a virus but should not be interpreted as true environmental concentrations [(19)](https://paperpile.com/c/rUn8Q0/F2Nb). Additionally, relative abundances estimated from bulk metagenomic samples shouldn’t be compared to enriched samples directly, even if they are from the same location or batch. Both sample types have their unique strengths and weaknesses (Step 0, Supplementary), including biases towards viruses that can be represented within that sample. Bulk metagenomic samples are challenging for assemblers as they usually contain a highly diverse viral community in front of a larger background signal from host cells (Step 3, Supplementary). This results in the ability to profile a broad community of viruses and their hosts at the cost of resolution. While enriched samples can display viral diversity at higher resolution due to a more balanced sequencing depth between species (resulting in less fragmented assemblies), they are limited to viruses that can be enriched, thus lacking host context and e.g. integrated proviruses. As such, both types of data are suitable for virome profiling, but which method to choose depends on the research question.

### **Step 12: Viral annotation and identification of AMGs**

Viruses often contain highly diverse AMGs [(77)](https://paperpile.com/c/rUn8Q0/Y6o9); genes of bacterial origin that encode for functions interacting with host metabolism [(78)](https://paperpile.com/c/rUn8Q0/RsyQ). Reported first in marine viruses [(79)](https://paperpile.com/c/rUn8Q0/jDLN), AMGs can enhance photosynthesis of cyanobacteria [(80)](https://paperpile.com/c/rUn8Q0/RSLy), aid energy metabolism in energy-limited ocean depths [(77)](https://paperpile.com/c/rUn8Q0/Y6o9), degrade herbicides in polluted soils [(81)](https://paperpile.com/c/rUn8Q0/CQc2), confer antibiotic resistance [(82)](https://paperpile.com/c/rUn8Q0/nYzc); and temperate viruses often contribute to host virulence [(83)](https://paperpile.com/c/rUn8Q0/B1KC). Additionally viral AMGs can modulate host metabolism in both lysogenic and lytic cycles, with lifestyle-associated functions being suggested [(78, 84, 85)](https://paperpile.com/c/rUn8Q0/pzvP+dKhZ+RsyQ). To understand the potential impact a virus can have on its host, viral genes need to first be predicted and annotated.

Gene prediction focuses on finding DNA features like protein-coding regions, non-protein-coding regions responsible for functional RNA molecules like tRNAs or rRNAs, and regulatory regions. Determining where to begin interpreting a raw DNA sequence poses a significant challenge, especially since viruses frequently contain overlapping genes [(86)](https://paperpile.com/c/rUn8Q0/8frw). Existing approaches can be divided into homology-based methods like **GeMoMa** ([(87)](https://paperpile.com/c/rUn8Q0/TvS7), no domain-restriction defined), which align the sequence to corresponding transcriptomic data or reference sequences, and *ab initio* methods like **GeneMarkS-2** ([(88)](https://paperpile.com/c/rUn8Q0/wgp7), bacteria/archaea), **Glimmer3** ([(89)](https://paperpile.com/c/rUn8Q0/cnNN), bacteria/archaea/viruses), **PHANOTATE** ([(90)](https://paperpile.com/c/rUn8Q0/nXfh), virus-exclusive) or **Prodigal** ([(91)](https://paperpile.com/c/rUn8Q0/br1a), bacteria/archaea, can handle viruses but not optimized), which interpret sequence patterns using mathematical models or others, relying on databases for model training [(92)](https://paperpile.com/c/rUn8Q0/oIj5).

Furthermore, functional annotation associates meaning to the predicted genes through alignments to references, or comparison with orthologous genes (e.g. **VOG database**, [(93)](https://paperpile.com/c/rUn8Q0/NWjS)), since orthologous genes in different species have a similar function, because they originate from the same gene of an ancestral species. However, both approaches are limited by the fact that many viral genes are of unknown function.

Since multiple tools are necessary to fully predict and annotate a genome, pipelines are a popular choice. **Prokka** [(94)](https://paperpile.com/c/rUn8Q0/MfU9) processes bacterial, archaeal, or viral DNA combining multiple gene prediction tools (including Prodigal). It annotates using several databases queried in order of size and expected hits, with user-provided protein references prioritized for speed. Custom pre-training with representative references to improve prediction accuracy is also supported.

**eggNOG-mapper** [(95)](https://paperpile.com/c/rUn8Q0/w5Uq) is a modular tool able to start analysis from (meta-)genomes, coding sequences, or proteins of all domains. Annotation is performed based on the **eggNOG** database [(96)](https://paperpile.com/c/rUn8Q0/ZKHE), which consists of orthologous groups from all domains. The results also include taxonomic information from the reference.

**Pharokka** [(97)](https://paperpile.com/c/rUn8Q0/9PPe) is a workflow tailored to prokaryotic viruses. It uses **PHANOTATE** (optionally **Prodigal**) for gene prediction and annotates genes using the **PHROGs** database [(98)](https://paperpile.com/c/rUn8Q0/6fnB). Additionally, antibiotic resistance genes and virulence factors commonly encoded by viruses are annotated using the **CARD** [(99)](https://paperpile.com/c/rUn8Q0/93YS) and **VFDB** [(100)](https://paperpile.com/c/rUn8Q0/DxAP) databases.

While not specifically tailored to viruses, **ABRicate** (T. Seemann, <https://github.com/tseemann/abricate>) screens DNA sequences for antimicrobial resistance genes and virulence factors using not only **CARD** and **VFDB** but also other eligible databases. Since input is annotated with a blast-based approach that provides hit coordinates, prior gene calling is not strictly necessary (nor provided).

**DRAM** [(101)](https://paperpile.com/c/rUn8Q0/Qk3a) specifically focuses on metabolism. Its viral mode **DRAM-v** requires contigs previously identified through **VirSorter/VirSorter2** [(51, 102)](https://paperpile.com/c/rUn8Q0/Cn2u+cWAE) and their associated files as input. It follows the DRAM pipeline but includes a comparison to **ViralRefSeq** [(103)](https://paperpile.com/c/rUn8Q0/W7vx). Additionally, DRAM-v includes rulesets applied to the annotated genes to obtain the auxiliary score, which measures the confidence that a gene is of viral origin, and several flags indicating AMG status or experimental verification.

Accurate gene prediction remains a bottleneck for correct gene annotations. The most common issues include incorrect start codon prediction and wrong gene boundary prediction leading to incomplete or merged genes [(104)](https://paperpile.com/c/rUn8Q0/SHik). Metagenomic datasets typically result in fragmented assemblies [(45)](https://paperpile.com/c/rUn8Q0/4n57) which complicate gene prediction at contig edges. Additionally, metagenomes can contain species using different genetic code translation tables (A. Elzanowski and J. Ostell, <https://www.ncbi.nlm.nih.gov/Taxonomy/Utils/wprintgc.cgi>), leading to incorrect gene predictions and annotations if the same code is applied throughout [(105)](https://paperpile.com/c/rUn8Q0/9k6v). These incorrect predictions propagate since public repositories commonly used for homology-based prediction, annotation, or model training frequently contain sequences with erroneous gene predictions [(106)](https://paperpile.com/c/rUn8Q0/lvAt).

Typically, annotations are validated manually or by referring to highly curated databases like **PHROGs**, **PDB** [(107)](https://paperpile.com/c/rUn8Q0/U5dr), or **UniProtKB/Swiss-Prot** [(108)](https://paperpile.com/c/rUn8Q0/CWZj), while interesting gene candidates are often confirmed experimentally as well (e.g. [(109)](https://paperpile.com/c/rUn8Q0/wwH7)). Alternatively, results can be improved by consulting transcriptome data to confirm gene predictions [(110)](https://paperpile.com/c/rUn8Q0/8J9g). However this is only applicable for expressed genes.

Besides labor-intensive manual validation and curation of gene predictions, there are tools aiming to aid in the recommended screening process. **GeneValidator** [(104)](https://paperpile.com/c/rUn8Q0/SHik) compares protein-coding gene predictions (DNA or amino acid) with provided or custom databases. Instead of annotating genes, it evaluates their quality based on e.g. reference gene length, coverage, and how conserved regions between query and references are (refer to [(111)](https://paperpile.com/c/rUn8Q0/6CHP) for guidance).

The constant increase of readily available references and machine learning alternatives for existing gene prediction and annotation methods is expected to lead to overall improvements [(92)](https://paperpile.com/c/rUn8Q0/oIj5). In particular, structure-based annotations are an emerging approach [(69, 112)](https://paperpile.com/c/rUn8Q0/snFw+1rVQ) based on the premise that viral proteins can be conserved at the structural level without showing any sequence homology [(113)](https://paperpile.com/c/rUn8Q0/EG16).

Refer to the following sources for details about the potential of viral AMGs [(78)](https://paperpile.com/c/rUn8Q0/RsyQ), RNA viral AMGs [(114)](https://paperpile.com/c/rUn8Q0/VQSB), current viral databases [(115)](https://paperpile.com/c/rUn8Q0/BGGa), gene prediction and annotation [(92, 116)](https://paperpile.com/c/rUn8Q0/oIj5+AGab), and benchmarking [(117)](https://paperpile.com/c/rUn8Q0/ULfx).

## References [Supplementary]

1. [Kieft K, Anantharaman K. 2022. Virus genomics: what is being overlooked? Curr Opin Virol 53:101200.](http://paperpile.com/b/rUn8Q0/MI85)

2. [Kleiner M, Hooper LV, Duerkop BA. 2015. Evaluation of methods to purify virus-like particles for metagenomic sequencing of intestinal viromes. BMC Genomics 16:7.](http://paperpile.com/b/rUn8Q0/hbo1)

3. [Thurber RV, Haynes M, Breitbart M, Wegley L, Rohwer F. 2009. Laboratory procedures to generate viral metagenomes. Nat Protoc 4:470–483.](http://paperpile.com/b/rUn8Q0/SWy1)

4. [Adams MH. 1949. THE STABILITY OF BACTERIAL VIRUSES IN SOLUTIONS OF SALTS. J Gen Physiol 32:579–594.](http://paperpile.com/b/rUn8Q0/Uhnp)

5. [Callanan J, Stockdale SR, Shkoporov A, Draper LA, Ross RP, Hill C. 2021. Biases in Viral Metagenomics-Based Detection, Cataloguing and Quantification of Bacteriophage Genomes in Human Faeces, a Review. Microorganisms 9:524.](http://paperpile.com/b/rUn8Q0/YI6D)

6. [Louten J. 2016. Chapter 2 - Virus Structure and Classification, p. 19–29. *In* Louten, J (ed.), Essential Human Virology. Academic Press, Boston.](http://paperpile.com/b/rUn8Q0/31K9)

7. [Hoyles L, McCartney AL, Neve H, Gibson GR, Sanderson JD, Heller KJ, van Sinderen D. 2014. Characterization of virus-like particles associated with the human faecal and caecal microbiota. Res Microbiol 165:803–812.](http://paperpile.com/b/rUn8Q0/jzRH)

8. [Klieve AV, Swain RA. 1993. Estimation of ruminal bacteriophage numbers by pulsed-field gel electrophoresis and laser densitometry. Applied and Environmental Microbiology 59:2299–2303.](http://paperpile.com/b/rUn8Q0/PEUJ)

9. [Ramachandran S, Larroche C, Pandey A. 2008. Production of Spores, p. 230–252. *In* Pandey, A, Soccol, CR, Larroche, C (eds.), Current Developments in Solid-state Fermentation. Springer, New York, NY.](http://paperpile.com/b/rUn8Q0/CMwI)

10. [Kumar A, Kodidela S, Tadrous E, Cory TJ, Walker CM, Smith AM, Mukherjee A, Kumar S. 2020. Extracellular Vesicles in Viral Replication and Pathogenesis and Their Potential Role in Therapeutic Intervention. Viruses 12:887.](http://paperpile.com/b/rUn8Q0/OMet)

11. [Kleiner M, Bushnell B, Sanderson KE, Hooper LV, Duerkop BA. 2020. Transductomics: sequencing-based detection and analysis of transduced DNA in pure cultures and microbial communities. Microbiome 8:158.](http://paperpile.com/b/rUn8Q0/8iPb)

12. [Cai L, Yang Y, Jiao N, Zhang R. 2015. Evaluation of Tangential Flow Filtration for the Concentration and Separation of Bacteria and Viruses in Contrasting Marine Environments. PLoS One 10:e0136741.](http://paperpile.com/b/rUn8Q0/oGET)

13. [Chen C, Guo L, Yang Y, Oguma K, Hou L-A. 2021. Comparative effectiveness of membrane technologies and disinfection methods for virus elimination in water: A review. Sci Total Environ 801:149678.](http://paperpile.com/b/rUn8Q0/Qtmz)

14. [d’Humières C, Touchon M, Dion S, Cury J, Ghozlane A, Garcia-Garcera M, Bouchier C, Ma L, Denamur E, P. C. Rocha E. 2019. A simple, reproducible and cost-effective procedure to analyse gut phageome: from phage isolation to bioinformatic approach. Sci Rep 9:11331.](http://paperpile.com/b/rUn8Q0/tqWW)

15. [Torii S, Oishi W, Zhu Y, Thakali O, Malla B, Yu Z, Zhao B, Arakawa C, Kitajima M, Hata A, Ihara M, Kyuwa S, Sano D, Haramoto E, Katayama H. 2021. Comparison of five polyethylene glycol precipitation procedures for the RT-qPCR based recovery of murine hepatitis virus, bacteriophage phi6, and pepper mild mottle virus as a surrogate for SARS-CoV-2 from wastewater. The Science of the Total Environment 807:150722.](http://paperpile.com/b/rUn8Q0/6vSd)

16. [Khan Mirzaei M, Xue J, Costa R, Ru J, Schulz S, Taranu ZE, Deng L. 2021. Challenges of Studying the Human Virome – Relevant Emerging Technologies. Trends Microbiol 29:171–181.](http://paperpile.com/b/rUn8Q0/Ry2W)

17. [Roux S, Solonenko NE, Dang VT, Poulos BT, Schwenck SM, Goldsmith DB, Coleman ML, Breitbart M, Sullivan MB. 2016. Towards quantitative viromics for both double-stranded and single-stranded DNA viruses. PeerJ 4:e2777.](http://paperpile.com/b/rUn8Q0/r9k3)

18. [Hegarty B, Riddell J V, Bastien E, Langenfeld K, Lindback M, Saini JS, Wing A, Zhang J, Duhaime M. 2024. Benchmarking informatics approaches for virus discovery: caution is needed when combining *in silico* identification methods. mSystems 9:e01105–23.](http://paperpile.com/b/rUn8Q0/CEs6)

19. [Roux S, Adriaenssens EM, Dutilh BE, Koonin EV, Kropinski AM, Krupovic M, Kuhn JH, Lavigne R, Brister JR, Varsani A, Amid C, Aziz RK, Bordenstein SR, Bork P, Breitbart M, Cochrane GR, Daly RA, Desnues C, Duhaime MB, Emerson JB, Enault F, Fuhrman JA, Hingamp P, Hugenholtz P, Hurwitz BL, Ivanova NN, Labonté JM, Lee K-B, Malmstrom RR, Martinez-Garcia M, Mizrachi IK, Ogata H, Páez-Espino D, Petit M-A, Putonti C, Rattei T, Reyes A, Rodriguez-Valera F, Rosario K, Schriml L, Schulz F, Steward GF, Sullivan MB, Sunagawa S, Suttle CA, Temperton B, Tringe SG, Thurber RV, Webster NS, Whiteson KL, Wilhelm SW, Wommack KE, Woyke T, Wrighton KC, Yilmaz P, Yoshida T, Young MJ, Yutin N, Allen LZ, Kyrpides NC, Eloe-Fadrosh EA. 2019. Minimum Information about an Uncultivated Virus Genome (MIUViG). Nat Biotechnol 37:29–37.](http://paperpile.com/b/rUn8Q0/F2Nb)

20. [Huang W, Khan Mirzaei M, Deng L. 2025. Comparative evaluation of long-term preservation methods for morphologically distinct bacteriophages. Microbiol Spectr 13:e01442–24.](http://paperpile.com/b/rUn8Q0/MxOS)

21. [Singh A, Yasheshwar, Kaushik NK, Kala D, Nagraik R, Gupta S, Kaushal A, Walia Y, Dhir S, Noorani MS. 2025. Conventional and cutting-edge advances in plant virus detection: emerging trends and techniques. 3 Biotech 15:100.](http://paperpile.com/b/rUn8Q0/QpD8)

22. [Cook R, Brown N, Rihtman B, Michniewski S, Redgwell T, Clokie M, Stekel DJ, Chen Y, Scanlan DJ, Hobman JL, Nelson A, Jones MA, Smith D, Millard A. 2024. The long and short of it: benchmarking viromics using Illumina, Nanopore and PacBio sequencing technologies. Microb Genom 10:001198.](http://paperpile.com/b/rUn8Q0/DzOo)

23. [Chen S, Zhou Y, Chen Y, Gu J. 2018. fastp: an ultra-fast all-in-one FASTQ preprocessor. Bioinformatics 34:i884–i890.](http://paperpile.com/b/rUn8Q0/16qp)

24. [Martin M. 2011. Cutadapt removes adapter sequences from high-throughput sequencing reads. EMBnet J 17:10–12.](http://paperpile.com/b/rUn8Q0/7Q7v)

25. [Bolger AM, Lohse M, Usadel B. 2014. Trimmomatic: a flexible trimmer for Illumina sequence data. Bioinformatics 30:2114–2120.](http://paperpile.com/b/rUn8Q0/WfpW)

26. [Altschul SF, Gish W, Miller W, Myers EW, Lipman DJ. 1990. Basic local alignment search tool. J Mol Biol 215:403–410.](http://paperpile.com/b/rUn8Q0/Gs31)

27. [Wood DE, Lu J, Langmead B. 2019. Improved metagenomic analysis with Kraken 2. Genome Biol 20:257.](http://paperpile.com/b/rUn8Q0/yuxt)

28. [Lu J, Breitwieser FP, Thielen P, Salzberg SL. 2017. Bracken: estimating species abundance in metagenomics data. PeerJ Comput Sci 3:e104.](http://paperpile.com/b/rUn8Q0/Yl03)

29. [Pinto Y, Chakraborty M, Jain N, Bhatt AS. 2024. Phage-inclusive profiling of human gut microbiomes with Phanta. Nat Biotechnol 42:651–662.](http://paperpile.com/b/rUn8Q0/a62V)

30. [Menzel P, Ng KL, Krogh A. 2016. Fast and sensitive taxonomic classification for metagenomics with Kaiju. Nat Commun 7:11257.](http://paperpile.com/b/rUn8Q0/TgEB)

31. [Burrows M, Wheeler D. 1994. A block sorting lossless data compression algorithm. Digital Equipment Corporation.](http://paperpile.com/b/rUn8Q0/fFAd)

32. [Langmead B, Salzberg SL. 2012. Fast gapped-read alignment with Bowtie 2. Nat Methods 9:357–359.](http://paperpile.com/b/rUn8Q0/6A1o)

33. [Li H, Durbin R. 2009. Fast and accurate short read alignment with Burrows–Wheeler transform. Bioinformatics 25:1754–1760.](http://paperpile.com/b/rUn8Q0/pFaP)

34. [Blanco-Míguez A, Beghini F, Cumbo F, McIver LJ, Thompson KN, Zolfo M, Manghi P, Dubois L, Huang KD, Thomas AM, Nickols WA, Piccinno G, Piperni E, Punčochář M, Valles-Colomer M, Tett A, Giordano F, Davies R, Wolf J, Berry SE, Spector TD, Franzosa EA, Pasolli E, Asnicar F, Huttenhower C, Segata N. 2023. Extending and improving metagenomic taxonomic profiling with uncharacterized species using MetaPhlAn 4. Nat Biotechnol 41:1633–1644.](http://paperpile.com/b/rUn8Q0/rAA9)

35. [Zolfo M, Pinto F, Asnicar F, Manghi P, Tett A, Bushman FD, Segata N. 2019. Detecting contamination in viromes using ViromeQC. Nature Biotechnology 37:1408–1412.](http://paperpile.com/b/rUn8Q0/MUVd)

36. [Van Uffelen A, Posadas A, Roosens NHC, Marchal K, De Keersmaecker SCJ, Vanneste K. 2024. Benchmarking bacterial taxonomic classification using nanopore metagenomics data of several mock communities. Sci Data 11:864.](http://paperpile.com/b/rUn8Q0/QzSt)

37. [Ghurye J, Treangen T, Fedarko M, Hervey WJ, Pop M. 2019. MetaCarvel: linking assembly graph motifs to biological variants. Genome Biol 20:174.](http://paperpile.com/b/rUn8Q0/6Urc)

38. [Nurk S, Meleshko D, Korobeynikov A, Pevzner PA. 2017. metaSPAdes: a new versatile metagenomic assembler. Genome Res 27:824–834.](http://paperpile.com/b/rUn8Q0/IJCG)

39. [Antipov D, Raiko M, Lapidus A, Pevzner PA. 2020. Metaviral SPAdes : assembly of viruses from metagenomic data. Bioinformatics 36:4126–4129.](http://paperpile.com/b/rUn8Q0/CAs9)

40. [Li D, Liu C-M, Luo R, Sadakane K, Lam T-W. 2015. MEGAHIT: an ultra-fast single-node solution for large and complex metagenomics assembly via succinct de Bruijn graph. Bioinformatics 31:1674–1676.](http://paperpile.com/b/rUn8Q0/DIks)

41. [Wick RR, Judd LM, Wyres KL, Holt KE. 2021. Recovery of small plasmid sequences via Oxford Nanopore sequencing. Microb Genom 7.](http://paperpile.com/b/rUn8Q0/zqw9)

42. [Shkoporov AN, Ryan FJ, Draper LA, Forde A, Stockdale SR, Daly KM, McDonnell SA, Nolan JA, Sutton TDS, Dalmasso M, McCann A, Ross RP, Hill C. 2018. Reproducible protocols for metagenomic analysis of human faecal phageomes. Microbiome 6:68.](http://paperpile.com/b/rUn8Q0/crXD)

43. [Gorzelak MA, Gill SK, Tasnim N, Ahmadi-Vand Z, Jay M, Gibson DL. 2015. Methods for Improving Human Gut Microbiome Data by Reducing Variability through Sample Processing and Storage of Stool. PLoS One 10:e0134802.](http://paperpile.com/b/rUn8Q0/vl1u)

44. [Sprouffske K, Aguilar-Rodríguez J, Wagner A. 2016. How Archiving by Freezing Affects the Genome-Scale Diversity of Escherichia coli Populations. Genome Biol Evol 8:1290–1298.](http://paperpile.com/b/rUn8Q0/NIh1)

45. [Sutton TDS, Clooney AG, Ryan FJ, Ross RP, Hill C. 2019. Choice of assembly software has a critical impact on virome characterisation. Microbiome 7:12.](http://paperpile.com/b/rUn8Q0/4n57)

46. [Kieft K, Zhou Z, Anantharaman K. 2020. VIBRANT: automated recovery, annotation and curation of microbial viruses, and evaluation of viral community function from genomic sequences. Microbiome 8:90.](http://paperpile.com/b/rUn8Q0/GCgL)

47. [Nayfach S, Camargo AP, Schulz F, Eloe-Fadrosh E, Roux S, Kyrpides NC. 2021. CheckV assesses the quality and completeness of metagenome-assembled viral genomes. Nat Biotechnol 39:578–585.](http://paperpile.com/b/rUn8Q0/vJ0m)

48. [Beaulaurier J, Luo E, Eppley JM, Uyl PD, Dai X, Burger A, Turner DJ, Pendelton M, Juul S, Harrington E, DeLong EF. 2020. Assembly-free single-molecule sequencing recovers complete virus genomes from natural microbial communities. Genome Res 30:437–446.](http://paperpile.com/b/rUn8Q0/OFkH)

49. [Leclercq S, Cordaux R. 2011. Do Phages Efficiently Shuttle Transposable Elements Among Prokaryotes? Evolution 65:3327–3331.](http://paperpile.com/b/rUn8Q0/go2P)

50. [Orakov A, Fullam A, Coelho LP, Khedkar S, Szklarczyk D, Mende DR, Schmidt TSB, Bork P. 2021. GUNC: detection of chimerism and contamination in prokaryotic genomes. Genome Biol 22:178.](http://paperpile.com/b/rUn8Q0/D1Ei)

51. [Guo J, Bolduc B, Zayed AA, Varsani A, Dominguez-Huerta G, Delmont TO, Pratama AA, Gazitúa MC, Vik D, Sullivan MB, Roux S. 2021. VirSorter2: a multi-classifier, expert-guided approach to detect diverse DNA and RNA viruses. Microbiome 9:37.](http://paperpile.com/b/rUn8Q0/cWAE)

52. [Camargo AP, Nayfach S, Chen I-MA, Palaniappan K, Ratner A, Chu K, Ritter SJ, Reddy TBK, Mukherjee S, Schulz F, Call L, Neches RY, Woyke T, Ivanova NN, Eloe-Fadrosh EA, Kyrpides NC, Roux S. 2023. IMG/VR v4: an expanded database of uncultivated virus genomes within a framework of extensive functional, taxonomic, and ecological metadata. Nucleic Acids Res 51:D733–D743.](http://paperpile.com/b/rUn8Q0/G5kS)

53. [Walker PJ, Siddell SG, Lefkowitz EJ, Mushegian AR, Adriaenssens EM, Alfenas-Zerbini P, Dempsey DM, Dutilh BE, García ML, Curtis Hendrickson R, Junglen S, Krupovic M, Kuhn JH, Lambert AJ, Łobocka M, Oksanen HM, Orton RJ, Robertson DL, Rubino L, Sabanadzovic S, Simmonds P, Smith DB, Suzuki N, Van Doorslaer K, Vandamme A-M, Varsani A, Zerbini FM. 2022. Recent changes to virus taxonomy ratified by the International Committee on Taxonomy of Viruses (2022). Arch Virol 167:2429–2440.](http://paperpile.com/b/rUn8Q0/vEbJ)

54. [Koonin EV, Kuhn JH, Dolja VV, Krupovic M. 2024. Megataxonomy and global ecology of the virosphere. ISME J 18:wrad042.](http://paperpile.com/b/rUn8Q0/1TGl)

55. [Krupovic M, Dolja VV, Koonin EV. 2019. Origin of viruses: primordial replicators recruiting capsids from hosts. Nat Rev Microbiol 17:449–458.](http://paperpile.com/b/rUn8Q0/e81e)

56. [Turner D, Kropinski AM, Adriaenssens EM. 2021. A Roadmap for Genome-Based Phage Taxonomy. Viruses 13:506.](http://paperpile.com/b/rUn8Q0/vnta)

57. [Simmonds P, Adams MJ, Benkő M, Breitbart M, Brister JR, Carstens EB, Davison AJ, Delwart E, Gorbalenya AE, Harrach B, Hull R, King AMQ, Koonin EV, Krupovic M, Kuhn JH, Lefkowitz EJ, Nibert ML, Orton R, Roossinck MJ, Sabanadzovic S, Sullivan MB, Suttle CA, Tesh RB, van der Vlugt RA, Varsani A, Zerbini FM. 2017. Virus taxonomy in the age of metagenomics. Nat Rev Microbiol 15:161–168.](http://paperpile.com/b/rUn8Q0/3tv6)

58. [Bin Jang H, Bolduc B, Zablocki O, Kuhn JH, Roux S, Adriaenssens EM, Brister JR, Kropinski AM, Krupovic M, Lavigne R, Turner D, Sullivan MB. 2019. Taxonomic assignment of uncultivated prokaryotic virus genomes is enabled by gene-sharing networks. Nat Biotechnol 37:632–639.](http://paperpile.com/b/rUn8Q0/3rEg)

59. [Nishimura Y, Yoshida T, Kuronishi M, Uehara H, Ogata H, Goto S. 2017. ViPTree: the viral proteomic tree server. Bioinformatics 33:2379–2380.](http://paperpile.com/b/rUn8Q0/e1ek)

60. [Meier-Kolthoff JP, Göker M. 2017. VICTOR: genome-based phylogeny and classification of prokaryotic viruses. Bioinformatics 33:3396–3404.](http://paperpile.com/b/rUn8Q0/kc6p)

61. [Mayne R, Aiewsakun P, Turner D, Adriaenssens EM, Simmonds P. 2024. GRAViTy-V2: a grounded viral taxonomy application. NAR Genom Bioinform 6:lqae183.](http://paperpile.com/b/rUn8Q0/9tUu)

62. [Moraru C, Varsani A, Kropinski AM. 2020. VIRIDIC—A Novel Tool to Calculate the Intergenomic Similarities of Prokaryote-Infecting Viruses. Viruses 12:1268.](http://paperpile.com/b/rUn8Q0/cGNY)

63. [Moraru C. 2023. VirClust—A Tool for Hierarchical Clustering, Core Protein Detection and Annotation of (Prokaryotic) Viruses. Viruses 15:1007.](http://paperpile.com/b/rUn8Q0/eUfy)

64. [Camargo AP, Roux S, Schulz F, Babinski M, Xu Y, Hu B, Chain PSG, Nayfach S, Kyrpides NC. 2024. Identification of mobile genetic elements with geNomad. Nat Biotechnol 42:1303–1312.](http://paperpile.com/b/rUn8Q0/18Qt)

65. [Steinegger M, Söding J. 2017. MMseqs2 enables sensitive protein sequence searching for the analysis of massive data sets. Nat Biotechnol 35:1026–1028.](http://paperpile.com/b/rUn8Q0/y4mN)

66. [Zheng K, Sun J, Liang Y, Kong L, Paez-Espino D, Mcminn A, Wang M. 2025. VITAP: a high precision tool for DNA and RNA viral classification based on meta-omic data. Nat Commun 16:2226.](http://paperpile.com/b/rUn8Q0/Pfg1)

67. [Zhou K, Kosmopoulos JC, Anantharaman K. 2024. vClassifier: a toolkit for species-level classification of prokaryotic viruses. bioRxiv https://doi.org/](http://paperpile.com/b/rUn8Q0/AE7K)[10.1101/2024.05.28.596318](http://dx.doi.org/10.1101/2024.05.28.596318)[.](http://paperpile.com/b/rUn8Q0/AE7K)

68. [Nasir A, Caetano-Anollés G. 2017. Identification of Capsid/Coat Related Protein Folds and Their Utility for Virus Classification. Front Microbiol 8.](http://paperpile.com/b/rUn8Q0/UlYQ)

69. [Mutz P, Camargo AP, Sahakyan H, Neri U, Butkovic A, Wolf YI, Krupovic M, Dolja VV, Koonin EV. 2024. The protein structurome of Orthornavirae and its dark matter. MBio 16:e03200–24.](http://paperpile.com/b/rUn8Q0/snFw)

70. [Kim D, Park S, Steinegger M. 2025. Unicore Enables Scalable and Accurate Phylogenetic Reconstruction with Structural Core Genes. Genome Biol Evol 17:evaf109.](http://paperpile.com/b/rUn8Q0/T4pC)

71. [Valencia-Toxqui G, Ramsey J. 2024. How to introduce a new bacteriophage on the block: a short guide to phage classification. J Virol 98:e01821–23.](http://paperpile.com/b/rUn8Q0/FLXd)

72. [Turner D, Adriaenssens EM, Lehman SM, Moraru C, Kropinski AM. 2024. Bacteriophage Taxonomy: A Continually Evolving Discipline, p. 27–45. *In* Azeredo, J, Sillankorva, S (eds.), Bacteriophage Therapy. Springer US, New York, NY.](http://paperpile.com/b/rUn8Q0/35N4)

73. [Simmonds P, Adriaenssens EM, Zerbini FM, Abrescia NGA, Aiewsakun P, Alfenas-Zerbini P, Bao Y, Barylski J, Drosten C, Duffy S, Duprex WP, Dutilh BE, Elena SF, García ML, Junglen S, Katzourakis A, Koonin EV, Krupovic M, Kuhn JH, Lambert AJ, Lefkowitz EJ, Łobocka M, Lood C, Mahony J, Meier-Kolthoff JP, Mushegian AR, Oksanen HM, Poranen MM, Reyes-Muñoz A, Robertson DL, Roux S, Rubino L, Sabanadzovic S, Siddell S, Skern T, Smith DB, Sullivan MB, Suzuki N, Turner D, Van Doorslaer K, Vandamme A-M, Varsani A, Vasilakis N. 2023. Four principles to establish a universal virus taxonomy. PLoS Biol 21:e3001922.](http://paperpile.com/b/rUn8Q0/rFkh)

74. [Grigson SR, Giles SK, Edwards RA, Papudeshi B. 2023. Knowing and Naming: Phage Annotation and Nomenclature for Phage Therapy. Clin Infect Dis 77:S352–S359.](http://paperpile.com/b/rUn8Q0/fcD3)

75. [Dutilh BE, Varsani A, Tong Y, Simmonds P, Sabanadzovic S, Rubino L, Roux S, Muñoz AR, Lood C, Lefkowitz EJ, Kuhn JH, Krupovic M, Edwards RA, Brister JR, Adriaenssens EM, Sullivan MB. 2021. Perspective on taxonomic classification of uncultivated viruses. Curr Opin Virol 51:207–215.](http://paperpile.com/b/rUn8Q0/FE4T)

76. [Aroney STN, Newell RJP, Nissen JN, Camargo AP, Tyson GW, Woodcroft BJ. 2025. CoverM: read alignment statistics for metagenomics. Bioinformatics 41:btaf147.](http://paperpile.com/b/rUn8Q0/hpCE)

77. [Coutinho FH, Rosselli R, Rodríguez-Valera F. 2019. Trends of Microdiversity Reveal Depth-Dependent Evolutionary Strategies of Viruses in the Mediterranean. mSystems 4.](http://paperpile.com/b/rUn8Q0/Y6o9)

78. [Sun M, Yuan S, Xia R, Ye M, Balcázar JL. 2023. Underexplored viral auxiliary metabolic genes in soil: Diversity and eco-evolutionary significance. Environ Microbiol 25:800–810.](http://paperpile.com/b/rUn8Q0/RsyQ)

79. [Breitbart M, Thompson LR, Suttle CA, Sullivan MB. 2007. Exploring the Vast Diversity of Marine Viruses. Oceanography (Wash D C) 20:135–139.](http://paperpile.com/b/rUn8Q0/jDLN)

80. [Fridman S, Flores-Uribe J, Larom S, Alalouf O, Liran O, Yacoby I, Salama F, Bailleul B, Rappaport F, Ziv T, Sharon I, Cornejo-Castillo FM, Philosof A, Dupont CL, Sánchez P, Acinas SG, Rohwer FL, Lindell D, Béjà O. 2017. A myovirus encoding both photosystem I and II proteins enhances cyclic electron flow in infected Prochlorococcus cells. Nat Microbiol 2:1350–1357.](http://paperpile.com/b/rUn8Q0/RSLy)

81. [Ghosh D, Roy K, Williamson KE, White DC, Wommack KE, Sublette KL, Radosevich M. 2008. Prevalence of Lysogeny among Soil Bacteria and Presence of 16S rRNA and *trzN* Genes in Viral-Community DNA. Appl Environ Microbiol 74:495–502.](http://paperpile.com/b/rUn8Q0/CQc2)

82. [Zhang Y, Guo Y, Qiu T, Gao M, Wang X. 2022. Bacteriophages: Underestimated vehicles of antibiotic resistance genes in the soil. Front Microbiol 13.](http://paperpile.com/b/rUn8Q0/nYzc)

83. [Fortier L-C, Sekulovic O. 2013. Importance of prophages to evolution and virulence of bacterial pathogens. Virulence 4:354–365.](http://paperpile.com/b/rUn8Q0/B1KC)

84. [Luo X-Q, Wang P, Li J-L, Ahmad M, Duan L, Yin L-Z, Deng Q-Q, Fang B-Z, Li S-H, Li W-J. 2022. Viral community-wide auxiliary metabolic genes differ by lifestyles, habitats, and hosts. Microbiome 10:190.](http://paperpile.com/b/rUn8Q0/pzvP)

85. [Tuttle MJ, Buchan A. 2020. Lysogeny in the oceans: Lessons from cultivated model systems and a reanalysis of its prevalence. Environ Microbiol 22:4919–4933.](http://paperpile.com/b/rUn8Q0/dKhZ)

86. [Schlub TE, Buchmann JP, Holmes EC. 2018. A Simple Method to Detect Candidate Overlapping Genes in Viruses Using Single Genome Sequences. Mol Biol Evol 35:2572–2581.](http://paperpile.com/b/rUn8Q0/8frw)

87. [Keilwagen J, Hartung F, Grau J. 2019. GeMoMa: Homology-Based Gene Prediction Utilizing Intron Position Conservation and RNA-seq Data, p. 161–177. *In* Kollmar, M (ed.), Gene Prediction: Methods and Protocols. Springer, New York, NY.](http://paperpile.com/b/rUn8Q0/TvS7)

88. [Lomsadze A, Gemayel K, Tang S, Borodovsky M. 2018. Modeling leaderless transcription and atypical genes results in more accurate gene prediction in prokaryotes. Genome Res 28:1079–1089.](http://paperpile.com/b/rUn8Q0/wgp7)

89. [Delcher AL, Bratke KA, Powers EC, Salzberg SL. 2007. Identifying bacterial genes and endosymbiont DNA with Glimmer. Bioinformatics 23:673–679.](http://paperpile.com/b/rUn8Q0/cnNN)

90. [McNair K, Zhou C, Dinsdale EA, Souza B, Edwards RA. 2019. PHANOTATE: a novel approach to gene identification in phage genomes. Bioinformatics 35:4537–4542.](http://paperpile.com/b/rUn8Q0/nXfh)

91. [Hyatt D, Chen G-L, LoCascio PF, Land ML, Larimer FW, Hauser LJ. 2010. Prodigal: prokaryotic gene recognition and translation initiation site identification. BMC Bioinformatics 11:119.](http://paperpile.com/b/rUn8Q0/br1a)

92. [Ejigu GF, Jung J. 2020. Review on the Computational Genome Annotation of Sequences Obtained by Next-Generation Sequencing. Biology (Basel) 9:295.](http://paperpile.com/b/rUn8Q0/oIj5)

93. [Trgovec-Greif L, Hellinger H-J, Mainguy J, Pfundner A, Frishman D, Kiening M, Webster NS, Laffy PW, Feichtinger M, Rattei T. 2024. VOGDB—Database of Virus Orthologous Groups. Viruses 16:1191.](http://paperpile.com/b/rUn8Q0/NWjS)

94. [Seemann T. 2014. Prokka: rapid prokaryotic genome annotation. Bioinformatics 30:2068–2069.](http://paperpile.com/b/rUn8Q0/MfU9)

95. [Cantalapiedra CP, Hernández-Plaza A, Letunic I, Bork P, Huerta-Cepas J. 2021. eggNOG-mapper v2: Functional Annotation, Orthology Assignments, and Domain Prediction at the Metagenomic Scale. Mol Biol Evol 38:5825–5829.](http://paperpile.com/b/rUn8Q0/w5Uq)

96. [Huerta-Cepas J, Szklarczyk D, Heller D, Hernández-Plaza A, Forslund SK, Cook H, Mende DR, Letunic I, Rattei T, Jensen LJ, von Mering C, Bork P. 2019. eggNOG 5.0: a hierarchical, functionally and phylogenetically annotated orthology resource based on 5090 organisms and 2502 viruses. Nucleic Acids Res 47:D309–D314.](http://paperpile.com/b/rUn8Q0/ZKHE)

97. [Bouras G, Nepal R, Houtak G, Psaltis AJ, Wormald P-J, Vreugde S. 2023. Pharokka: a fast scalable bacteriophage annotation tool. Bioinformatics 39:btac776.](http://paperpile.com/b/rUn8Q0/9PPe)

98. [Terzian P, Olo Ndela E, Galiez C, Lossouarn J, Pérez Bucio RE, Mom R, Toussaint A, Petit M-A, Enault F. 2021. PHROG: families of prokaryotic virus proteins clustered using remote homology. NAR Genom Bioinform 3:lqab067.](http://paperpile.com/b/rUn8Q0/6fnB)

99. [Alcock BP, Raphenya AR, Lau TTY, Tsang KK, Bouchard M, Edalatmand A, Huynh W, Nguyen A-LV, Cheng AA, Liu S, Min SY, Miroshnichenko A, Tran H-K, Werfalli RE, Nasir JA, Oloni M, Speicher DJ, Florescu A, Singh B, Faltyn M, Hernandez-Koutoucheva A, Sharma AN, Bordeleau E, Pawlowski AC, Zubyk HL, Dooley D, Griffiths E, Maguire F, Winsor GL, Beiko RG, Brinkman FSL, Hsiao WWL, Domselaar GV, McArthur AG. 2020. CARD 2020: antibiotic resistome surveillance with the comprehensive antibiotic resistance database. Nucleic Acids Res 48:D517–D525.](http://paperpile.com/b/rUn8Q0/93YS)

100. [Liu B, Zheng D, Jin Q, Chen L, Yang J. 2019. VFDB 2019: a comparative pathogenomic platform with an interactive web interface. Nucleic Acids Res 47:D687–D692.](http://paperpile.com/b/rUn8Q0/DxAP)

101. [Shaffer M, Borton MA, McGivern BB, Zayed AA, La Rosa SL, Solden LM, Liu P, Narrowe AB, Rodríguez-Ramos J, Bolduc B, Gazitúa MC, Daly RA, Smith GJ, Vik DR, Pope PB, Sullivan MB, Roux S, Wrighton KC. 2020. DRAM for distilling microbial metabolism to automate the curation of microbiome function. Nucleic Acids Res 48:8883–8900.](http://paperpile.com/b/rUn8Q0/Qk3a)

102. [Roux S, Enault F, Hurwitz BL, Sullivan MB. 2015. VirSorter: mining viral signal from microbial genomic data. PeerJ 3:e985.](http://paperpile.com/b/rUn8Q0/Cn2u)

103. [Goldfarb T, Kodali VK, Pujar S, Brover V, Robbertse B, Farrell CM, Oh D-H, Astashyn A, Ermolaeva O, Haddad D, Hlavina W, Hoffman J, Jackson JD, Joardar VS, Kristensen D, Masterson P, McGarvey KM, McVeigh R, Mozes E, Murphy MR, Schafer SS, Souvorov A, Spurrier B, Strope PK, Sun H, Vatsan AR, Wallin C, Webb D, Brister JR, Hatcher E, Kimchi A, Klimke W, Marchler-Bauer A, Pruitt KD, Thibaud-Nissen F, Murphy TD. 2025. NCBI RefSeq: reference sequence standards through 25 years of curation and annotation. Nucleic Acids Res 53:D243–D257.](http://paperpile.com/b/rUn8Q0/W7vx)

104. [Drăgan M-A, Moghul I, Priyam A, Bustos C, Wurm Y. 2016. GeneValidator: identify problems with protein-coding gene predictions. Bioinformatics 32:1559–1561.](http://paperpile.com/b/rUn8Q0/SHik)

105. [Schmitz MA, Dimonaco NJ, Clavel T, Hitch TCA. 2025. Lineage-specific microbial protein prediction enables large-scale exploration of protein ecology within the human gut. Nat Commun 16:3204.](http://paperpile.com/b/rUn8Q0/9k6v)

106. [Meyer C, Scalzitti N, Jeannin-Girardon A, Collet P, Poch O, Thompson JD. 2020. Understanding the causes of errors in eukaryotic protein-coding gene prediction: a case study of primate proteomes. BMC Bioinformatics 21:513.](http://paperpile.com/b/rUn8Q0/lvAt)

107. [Berman HM, Westbrook J, Feng Z, Gilliland G, Bhat TN, Weissig H, Shindyalov IN, Bourne PE. 2000. The Protein Data Bank. Nucleic Acids Res 28:235–242.](http://paperpile.com/b/rUn8Q0/U5dr)

108. [The UniProt Consortium. 2025. UniProt: the Universal Protein Knowledgebase in 2025. Nucleic Acids Res 53:D609–D617.](http://paperpile.com/b/rUn8Q0/CWZj)

109. [Gehlert FO, Weidenbach K, Barüske B, Hallack D, Repnik U, Schmitz RA. 2023. Newly Established Genetic System for Functional Analysis of MetSV. Int J Mol Sci 24:11163.](http://paperpile.com/b/rUn8Q0/wwH7)

110. [Keilwagen J, Hartung F, Paulini M, Twardziok SO, Grau J. 2018. Combining RNA-seq data and homology-based gene prediction for plants, animals and fungi. BMC Bioinformatics 19:189.](http://paperpile.com/b/rUn8Q0/8J9g)

111. [Moghul I, Priyam A, Wurm Y. 2019. Choosing the Best Gene Predictions with GeneValidator, p. 257–267. *In* Kollmar, M (ed.), Gene Prediction: Methods and Protocols. Springer, New York, NY.](http://paperpile.com/b/rUn8Q0/6CHP)

112. [Longin H, Bouras G, Grigson SR, Edwards RA, Hendrix H, Lavigne R, van Noort V. 2025. Fold first, ask later: structure-informed function annotation of Pseudomonas phage proteins. bioRxiv https://doi.org/](http://paperpile.com/b/rUn8Q0/1rVQ)[10.1101/2025.07.17.665397](http://dx.doi.org/10.1101/2025.07.17.665397)[.](http://paperpile.com/b/rUn8Q0/1rVQ)

113. [Dion MB, Oechslin F, Moineau S. 2020. Phage diversity, genomics and phylogeny. Nat Rev Microbiol 18:125–138.](http://paperpile.com/b/rUn8Q0/EG16)

114. [Zhao Y, Zhang Z, Feng M, Wen R, Liu P. 2025. Functional and evolutionary characterization of potential auxiliary metabolic genes of the global RNA virome. iMetaOmics 2:e70002.](http://paperpile.com/b/rUn8Q0/VQSB)

115. [Ritsch M, Cassman NA, Saghaei S, Marz M. 2023. Navigating the Landscape: A Comprehensive Review of Current Virus Databases. Viruses 15:1834.](http://paperpile.com/b/rUn8Q0/BGGa)

116. [Grigson SR, Bouras G, Dutilh BE, Olson RD, Edwards RA. 2025. Computational function prediction of bacteria and phage proteins. Microbiol Mol Biol Rev e0002225.](http://paperpile.com/b/rUn8Q0/AGab)

117. [González-Tortuero E, Krishnamurthi R, Allison HE, Goodhead IB, James CE. 2021. Comparative analysis of gene prediction tools for viral genome annotation. bioRxiv https://doi.org/](http://paperpile.com/b/rUn8Q0/ULfx)[10.1101/2021.12.11.472104](http://dx.doi.org/10.1101/2021.12.11.472104)[.](http://paperpile.com/b/rUn8Q0/ULfx)

118. [Ren J, Song K, Deng C, Ahlgren NA, Fuhrman JA, Li Y, Xie X, Poplin R, Sun F. 2020. Identifying viruses from metagenomic data using deep learning. Quant Biol 8:64–77.](http://paperpile.com/b/rUn8Q0/jCIA)

119. [Jurtz VI, Villarroel J, Lund O, Larsen MV, Nielsen M. 2016. MetaPhinder—Identifying Bacteriophage Sequences in Metagenomic Data Sets. PLoS One 11:e0163111.](http://paperpile.com/b/rUn8Q0/sbat)

120. [Starikova EV, Tikhonova PO, Prianichnikov NA, Rands CM, Zdobnov EM, Ilina EN, Govorun VM. 2020. Phigaro: high-throughput prophage sequence annotation. Bioinformatics 36:3882–3884.](http://paperpile.com/b/rUn8Q0/gQv1)

121. [Fang Z, Tan J, Wu S, Li M, Xu C, Xie Z, Zhu H. 2019. PPR-Meta: a tool for identifying phages and plasmids from metagenomic fragments using deep learning. Gigascience 8:giz066.](http://paperpile.com/b/rUn8Q0/hSCV)

122. [Auslander N, Gussow AB, Benler S, Wolf YI, Koonin EV. 2020. Seeker: alignment-free identification of bacteriophage genomes by deep learning. Nucleic Acids Res 48:e121.](http://paperpile.com/b/rUn8Q0/1fjv)

123. [Ren J, Ahlgren NA, Lu YY, Fuhrman JA, Sun F. 2017. VirFinder: a novel k-mer based tool for identifying viral sequences from assembled metagenomic data. Microbiome 5.](http://paperpile.com/b/rUn8Q0/1Z0C)

124. [Olm MR, Brown CT, Brooks B, Banfield JF. 2017. dRep: a tool for fast and accurate genomic comparisons that enables improved genome recovery from metagenomes through de-replication. ISME J 11:2864–2868.](http://paperpile.com/b/rUn8Q0/IT61)

125. [Jain C, Rodriguez-R LM, Phillippy AM, Konstantinidis KT, Aluru S. 2018. High throughput ANI analysis of 90K prokaryotic genomes reveals clear species boundaries. Nat Commun 9:5114.](http://paperpile.com/b/rUn8Q0/P2A3)

126. [Zielezinski A, Gudyś A, Barylski J, Siminski K, Rozwalak P, Dutilh BE, Deorowicz S. 2025. Ultrafast and accurate sequence alignment and clustering of viral genomes. Nat Methods 22:1191–1194.](http://paperpile.com/b/rUn8Q0/g6g8)

127. [Ondov BD, Treangen TJ, Melsted P, Mallonee AB, Bergman NH, Koren S, Phillippy AM. 2016. Mash: fast genome and metagenome distance estimation using MinHash. Genome Biol 17:132.](http://paperpile.com/b/rUn8Q0/fnC5)

128. [Chen L, Banfield JF. 2024. COBRA improves the completeness and contiguity of viral genomes assembled from metagenomes. Nat Microbiol 9:737–750.](http://paperpile.com/b/rUn8Q0/cG8v)

129. [Arisdakessian CG, Nigro OD, Steward GF, Poisson G, Belcaid M. 2021. CoCoNet: an efficient deep learning tool for viral metagenome binning. Bioinformatics 37:2803–2810.](http://paperpile.com/b/rUn8Q0/gOxS)

130. [Kang DD, Li F, Kirton E, Thomas A, Egan R, An H, Wang Z. 2019. MetaBAT 2: an adaptive binning algorithm for robust and efficient genome reconstruction from metagenome assemblies. PeerJ 7:e7359.](http://paperpile.com/b/rUn8Q0/HrEP)

131. [Johansen J, Plichta DR, Nissen JN, Jespersen ML, Shah SA, Deng L, Stokholm J, Bisgaard H, Nielsen DS, Sørensen SJ, Rasmussen S. 2022. Genome binning of viral entities from bulk metagenomics data. Nat Commun 13:965.](http://paperpile.com/b/rUn8Q0/oUc5)

132. [Nissen JN, Johansen J, Allesøe RL, Sønderby CK, Armenteros JJA, Grønbech CH, Jensen LJ, Nielsen HB, Petersen TN, Winther O, Rasmussen S. 2021. Improved metagenome binning and assembly using deep variational autoencoders. Nat Biotechnol 39:555–560.](http://paperpile.com/b/rUn8Q0/O6RL)

133. [Kieft K, Adams A, Salamzade R, Kalan L, Anantharaman K. 2022. vRhyme enables binning of viral genomes from metagenomes. Nucleic Acids Res 50:e83.](http://paperpile.com/b/rUn8Q0/rxUw)

134. [Hockenberry AJ, Wilke CO. 2021. BACPHLIP: predicting bacteriophage lifestyle from conserved protein domains. PeerJ 9:e11396.](http://paperpile.com/b/rUn8Q0/10yr)

135. [Zhang Y, Mao M, Zhang R, Liao Y-T, Wu VCH. 2024. DeepPL: A deep-learning-based tool for the prediction of bacteriophage lifecycle. PLoS Comput Biol 20:e1012525.](http://paperpile.com/b/rUn8Q0/vBm7)

136. [Wu S, Fang Z, Tan J, Li M, Wang C, Guo Q, Xu C, Jiang X, Zhu H. 2021. DeePhage: distinguishing virulent and temperate phage-derived sequences in metavirome data with a deep learning approach. Gigascience 10:giab056.](http://paperpile.com/b/rUn8Q0/5kDg)

137. [Shang J, Tang X, Sun Y. 2023. PhaTYP: predicting the lifestyle for bacteriophages using BERT. Brief Bioinform 24:bbac487.](http://paperpile.com/b/rUn8Q0/4Dln)

138. [Shang J, Peng C, Liao H, Tang X, Sun Y. 2023. PhaBOX: a web server for identifying and characterizing phage contigs in metagenomic data. Bioinform Adv 3:vbad101.](http://paperpile.com/b/rUn8Q0/74St)

139. [Kieft K, Anantharaman K. 2022. Deciphering Active Prophages from Metagenomes. mSystems 7:e00084–22.](http://paperpile.com/b/rUn8Q0/2tOC)

140. [Peng X, Ru J, Mirzaei MK, Deng L. 2022. Replidec - Use naive Bayes classifier to identify virus lifecycle from metagenomics data. bioRxiv https://doi.org/](http://paperpile.com/b/rUn8Q0/n4wh)[10.1101/2022.07.18.500415](http://dx.doi.org/10.1101/2022.07.18.500415)[.](http://paperpile.com/b/rUn8Q0/n4wh)

141. [Dion MB, Plante P-L, Zufferey E, Shah SA, Corbeil J, Moineau S. 2021. Streamlining CRISPR spacer-based bacterial host predictions to decipher the viral dark matter. Nucleic Acids Res 49:3127–3138.](http://paperpile.com/b/rUn8Q0/RoWg)

142. [Roux S, Camargo AP, Coutinho FH, Dabdoub SM, Dutilh BE, Nayfach S, Tritt A. 2023. iPHoP: An integrated machine learning framework to maximize host prediction for metagenome-derived viruses of archaea and bacteria. PLoS Biol 21:e3002083.](http://paperpile.com/b/rUn8Q0/9BH9)

143. [Lu C, Zhang Z, Cai Z, Zhu Z, Qiu Y, Wu A, Jiang T, Zheng H, Peng Y. 2021. Prokaryotic virus host predictor: a Gaussian model for host prediction of prokaryotic viruses in metagenomics. BMC Biol 19:5.](http://paperpile.com/b/rUn8Q0/vHFg)

144. [Coutinho FH, Zaragoza-Solas A, López-Pérez M, Barylski J, Zielezinski A, Dutilh BE, Edwards R, Rodriguez-Valera F. 2021. RaFAH: Host prediction for viruses of Bacteria and Archaea based on protein content. Patterns (N Y) 2.](http://paperpile.com/b/rUn8Q0/Zxps)

145. [Zhang R, Mirdita M, Levy Karin E, Norroy C, Galiez C, Söding J. 2021. SpacePHARER: sensitive identification of phages from CRISPR spacers in prokaryotic hosts. Bioinformatics 37:3364–3366.](http://paperpile.com/b/rUn8Q0/j49u)

146. [Ahlgren NA, Ren J, Lu YY, Fuhrman JA, Sun F. 2017. Alignment-free d_2^* oligonucleotide frequency dissimilarity measure improves prediction of hosts from metagenomically-derived viral sequences. Nucleic Acids Res 45:39–53.](http://paperpile.com/b/rUn8Q0/swzK)

147. [Galiez C, Siebert M, Enault F, Vincent J, Söding J. 2017. WIsH: who is the host? Predicting prokaryotic hosts from metagenomic phage contigs. Bioinformatics 33:3113–3114.](http://paperpile.com/b/rUn8Q0/JVnq)

148. [Yan Y, Zheng J, Zhang X, Yin Y. 2023. dbAPIS: a database of anti-prokaryotic immune system genes. Nucleic Acids Res 52:D419–D425.](http://paperpile.com/b/rUn8Q0/erGF)

149. [Hou S, Tang T, Cheng S, Liu Y, Xia T, Chen T, Fuhrman JA, Sun F. 2024. DeepMicroClass sorts metagenomic contigs into prokaryotes, eukaryotes and viruses. NAR Genom Bioinform 6:lqae044.](http://paperpile.com/b/rUn8Q0/UHqP)

150. [Gauthier CH, Abad L, Venbakkam AK, Malnak J, Russell DA, Hatfull GF. 2022. DEPhT: a novel approach for efficient prophage discovery and precise extraction. Nucleic Acids Res 50:e75.](http://paperpile.com/b/rUn8Q0/H9YC)

151. [Vik DR, Roux S, Brum JR, Bolduc B, Emerson JB, Padilla CC, Stewart FJ, Sullivan MB. 2017. Putative archaeal viruses from the mesopelagic ocean. PeerJ 5:e3428.](http://paperpile.com/b/rUn8Q0/kkPH)

152. [Vik D, Bolduc B, Roux S, Sun CL, Pratama AA, Krupovic M, Sullivan MB. 2023. MArVD2: a machine learning enhanced tool to discriminate between archaeal and bacterial viruses in viral datasets. ISME Commun 3:87.](http://paperpile.com/b/rUn8Q0/lG98)

153. [Amgarten D, Braga LPP, da Silva AM, Setubal JC. 2018. MARVEL, a Tool for Prediction of Bacteriophage Sequences in Metagenomic Bins. Front Genet 9:304.](http://paperpile.com/b/rUn8Q0/tKf6)

154. [Arndt D, Grant JR, Marcu A, Sajed T, Pon A, Liang Y, Wishart DS. 2016. PHASTER: a better, faster version of the PHAST phage search tool. Nucleic Acids Res 44:W16–21.](http://paperpile.com/b/rUn8Q0/8tCZ)

155. [Wishart DS, Han S, Saha S, Oler E, Peters H, Grant JR, Stothard P, Gautam V. 2023. PHASTEST: faster than PHASTER, better than PHAST. Nucleic Acids Res 51:W443–W450.](http://paperpile.com/b/rUn8Q0/uVun)

156. [Copeland CJ, Roddy JW, Schmidt AK, Secor PR, Wheeler TJ. 2024. VIBES: a workflow for annotating and visualizing viral sequences integrated into bacterial genomes. NAR Genom Bioinform 6:lqae030.](http://paperpile.com/b/rUn8Q0/fx9d)

157. [Tampuu A, Bzhalava Z, Dillner J, Vicente R. 2019. ViraMiner: Deep learning on raw DNA sequences for identifying viral genomes in human samples. PLoS One 14:e0222271.](http://paperpile.com/b/rUn8Q0/Md0o)

158. [Garretto A, Hatzopoulos T, Putonti C. 2019. virMine: automated detection of viral sequences from complex metagenomic samples. PeerJ 7:e6695.](http://paperpile.com/b/rUn8Q0/Swqo)

159. [Johnson G, Putonti C. 2022. virMine 2.0: Identifying Viral Sequences in Microbial Communities. Microbiol Resour Announc 11:e0010722.](http://paperpile.com/b/rUn8Q0/tdEp)

160. [Zheng T, Li J, Ni Y, Kang K, Misiakou M-A, Imamovic L, Chow BKC, Rode AA, Bytzer P, Sommer M, Panagiotou G. 2019. Mining, analyzing, and integrating viral signals from metagenomic data. Microbiome 7:42.](http://paperpile.com/b/rUn8Q0/Nk80)

161. [Fu L, Niu B, Zhu Z, Wu S, Li W. 2012. CD-HIT: accelerated for clustering the next-generation sequencing data. Bioinformatics 28:3150–3152.](http://paperpile.com/b/rUn8Q0/CmWI)

162. [Mallawaarachchi V, Roach MJ, Decewicz P, Papudeshi B, Giles SK, Grigson SR, Bouras G, Hesse RD, Inglis LK, Hutton ALK, Dinsdale EA, Edwards RA. 2023. Phables: from fragmented assemblies to high-quality bacteriophage genomes. Bioinformatics 39.](http://paperpile.com/b/rUn8Q0/Og4B)

163. [McNair K, Bailey BA, Edwards RA. 2012. PHACTS, a computational approach to classifying the lifestyle of phages. Bioinformatics 28:614–618.](http://paperpile.com/b/rUn8Q0/m9AX)

164. [Pan Y, Gao H, Lin H, Liu Z, Tang L, Li S. 2018. Identification of Bacteriophage Virion Proteins Using Multinomial Naïve Bayes with g-Gap Feature Tree. Int J Mol Sci 19.](http://paperpile.com/b/rUn8Q0/YXkR)

165. [Aiewsakun P, Simmonds P. 2018. The genomic underpinnings of eukaryotic virus taxonomy: creating a sequence-based framework for family-level virus classification. Microbiome 6:38.](http://paperpile.com/b/rUn8Q0/MA8Y)

166. [Shang J, Jiang J, Sun Y. 2021. Bacteriophage classification for assembled contigs using graph convolutional network. Bioinformatics 37:i25–i33.](http://paperpile.com/b/rUn8Q0/bnN9)

167. [Jiang J-Z, Yuan W-G, Shang J, Shi Y-H, Yang L-L, Liu M, Zhu P, Jin T, Sun Y, Yuan L-H. 2023. Virus classification for viral genomic fragments using PhaGCN2. Brief Bioinform 24.](http://paperpile.com/b/rUn8Q0/DfLZ)

168. [Guan J, Peng C, Shang J, Tang X, Sun Y. 2023. PhaGenus: genus-level classification of bacteriophages using a Transformer model. Brief Bioinform 24.](http://paperpile.com/b/rUn8Q0/Zz46)

169. [Millard A, Denise R, Lestido M, Thomas MT, Webster D, Turner D, Sicheritz-Pontén T. 2025. taxMyPhage: Automated Taxonomy of dsDNA Phage Genomes at the Genus and Species Level. Phage (New Rochelle) 6:5–11.](http://paperpile.com/b/rUn8Q0/VJjx)

170. [Zhang 张韬 T, Liu 刘依云 Y, Guo 郭栩彤 X, Zhang 张欣然 X, Zheng 郑欣畅 X, Zhang 张陌尘 M, Bao 鲍一明 Y. 2025. VISTA: A tool for fast taxonomic assignment of viral genome sequences. Genomics Proteomics Bioinformatics 23.](http://paperpile.com/b/rUn8Q0/YRlF)

171. [Pons JC, Paez-Espino D, Riera G, Ivanova N, Kyrpides NC, Llabrés M. 2021. VPF-Class: taxonomic assignment and host prediction of uncultivated viruses based on viral protein families. Bioinformatics 37:1805–1813.](http://paperpile.com/b/rUn8Q0/yA1p)

172. [Birolo G, Telatin A. 2022. BamToCov: an efficient toolkit for sequence coverage calculations. Bioinformatics 38:2617–2618.](http://paperpile.com/b/rUn8Q0/vOSW)

173. [Quinlan AR. 2014. BEDTools: The Swiss-Army Tool for Genome Feature Analysis. Curr Protoc Bioinformatics 47:11.12.1–34.](http://paperpile.com/b/rUn8Q0/pyU4)

174. [Danecek P, Bonfield JK, Liddle J, Marshall J, Ohan V, Pollard MO, Whitwham A, Keane T, McCarthy SA, Davies RM, Li H. 2021. Twelve years of SAMtools and BCFtools. Gigascience 10.](http://paperpile.com/b/rUn8Q0/C3NW)

175. [Wei T, Lu C, Du H, Yang Q, Qi X, Liu Y, Zhang Y, Chen C, Li Y, Tang Y, Zhang W-H, Tao X, Jiang N. 2024. DeepPBI-KG: a deep learning method for the prediction of phage-bacteria interactions based on key genes. Brief Bioinform 25.](http://paperpile.com/b/rUn8Q0/Ksn4)

176. [Kinsella CM, Deijs M, Becker C, Broekhuizen P, van Gool T, Bart A, Schaefer AS, van der Hoek L. 2022. Host prediction for disease-associated gastrointestinal cressdnaviruses. Virus Evol 8:veac087.](http://paperpile.com/b/rUn8Q0/e0DP)

177. [Baláž A, Kajsik M, Budiš J, Szemes T, Turňa J. 2023. PHERI-Phage Host ExploRation Pipeline. Microorganisms 11.](http://paperpile.com/b/rUn8Q0/Jifn)

178. [Zhou F, Gan R, Zhang F, Ren C, Yu L, Si Y, Huang Z. 2022. PHISDetector: A Tool to Detect Diverse In Silico Phage-host Interaction Signals for Virome Studies. Genomics Proteomics Bioinformatics 20:508–523.](http://paperpile.com/b/rUn8Q0/i83M)

179. [Zielezinski A, Deorowicz S, Gudyś A. 2022. PHIST: fast and accurate prediction of prokaryotic hosts from metagenomic viral sequences. Bioinformatics 38:1447–1449.](http://paperpile.com/b/rUn8Q0/KlEi)

180. [Bastien GE, Cable RN, Batterbee C, Wing AJ, Zaman L, Duhaime MB. 2024. Virus-host interactions predictor (VHIP): Machine learning approach to resolve microbial virus-host interaction networks. PLoS Comput Biol 20:e1011649.](http://paperpile.com/b/rUn8Q0/lhZp)

181. [Wang W, Ren J, Tang K, Dart E, Ignacio-Espinoza JC, Fuhrman JA, Braun J, Sun F, Ahlgren NA. 2020. A network-based integrated framework for predicting virus-prokaryote interactions. NAR Genom Bioinform 2:lqaa044.](http://paperpile.com/b/rUn8Q0/eHrL)

182. [Ecale Zhou CL, Malfatti S, Kimbrel J, Philipson C, McNair K, Hamilton T, Edwards R, Souza B. 2019. multiPhATE: bioinformatics pipeline for functional annotation of phage isolates. Bioinformatics 35:4402–4404.](http://paperpile.com/b/rUn8Q0/brkd)

183. [Ecale Zhou CL, Kimbrel J, Edwards R, McNair K, Souza BA, Malfatti S. 2021. MultiPhATE2: code for functional annotation and comparison of phage genomes. G3 (Bethesda) 11.](http://paperpile.com/b/rUn8Q0/Uc5F)

184. [Bouras G, Grigson SR, Mirdita M, Heinzinger M, Papudeshi B, Mallawaarachchi V, Green R, Kim RS, Mihalia V, Psaltis AJ, Wormald P-J, Vreugde S, Steinegger M, Edwards RA. 2025. Protein Structure Informed Bacteriophage Genome Annotation with Phold. bioRxiv https://doi.org/](http://paperpile.com/b/rUn8Q0/jWnn)[10.1101/2025.08.05.668817](http://dx.doi.org/10.1101/2025.08.05.668817)[.](http://paperpile.com/b/rUn8Q0/jWnn)

185. [Grigson S, Mallawaarachchi V, Roach, M. R., Papudeshi, B., Bouras, G., Decewicz, P., Dinsdale, E. A. & Edwards, R. A. 2023. susiegriggo/Phynteny: Phynteny 0.1.10. Zenodo.](http://paperpile.com/b/rUn8Q0/KrEm) <http://dx.doi.org/10.5281/ZENODO.8128917>[.](http://paperpile.com/b/rUn8Q0/KrEm)

186. [Kim RS, Levy Karin E, Mirdita M, Chikhi R, Steinegger M. 2025. BFVD-a large repository of predicted viral protein structures. Nucleic Acids Res 53:D340–D347.](http://paperpile.com/b/rUn8Q0/bO57)

187. [Zayed AA, Lücking D, Mohssen M, Cronin D, Bolduc B, Gregory AC, Hargreaves KR, Piehowski PD, White RA Iii, Huang EL, Adkins JN, Roux S, Moraru C, Sullivan MB. 2021. efam: an expanded, metaproteome-supported HMM profile database of viral protein families. Bioinformatics 37:4202–4208.](http://paperpile.com/b/rUn8Q0/gBNr)

188. [Grazziotin AL, Koonin EV, Kristensen DM. 2017. Prokaryotic Virus Orthologous Groups (pVOGs): a resource for comparative genomics and protein family annotation. Nucleic Acids Res 45:D491–D498.](http://paperpile.com/b/rUn8Q0/eM9N)

189. [Skewes-Cox P, Sharpton TJ, Pollard KS, DeRisi JL. 2014. Profile hidden Markov models for the detection of viruses within metagenomic sequence data. PLoS One 9:e105067.](http://paperpile.com/b/rUn8Q0/vbnb)

190. [Moreno-Gallego JL, Reyes A. 2021. Informative Regions In Viral Genomes. Viruses 13:1164.](http://paperpile.com/b/rUn8Q0/5TAM)

191. [Adriaenssens EM, Roux S, Brister JR, Karsch-Mizrachi I, Kuhn JH, Varsani A, Yigang T, Reyes A, Lood C, Lefkowitz EJ, Sullivan MB, Edwards RA, Simmonds P, Rubino L, Sabanadzovic S, Krupovic M, Dutilh BE. 2023. Guidelines for public database submission of uncultivated virus genome sequences for taxonomic classification. Nature Biotechnology 41:898–902.](http://paperpile.com/b/rUn8Q0/OXAH)
